# From Filamentary Failure to Durable Halide Perovskite Memristors

**DOI:** 10.1021/acsnano.6c04620

**Published:** 2026-08-05

**Authors:** Tuo Hu, Nathalie Tuazon, Zhaojian Xu, Sunney Gao, Jisu Hong, Hussein Badran, Ross A. Kerner, Qiangfei Xia, Barry P. Rand

**Affiliations:** † Department of Electrical and Computer Engineering, 6740 Princeton University, Princeton, New Jersey 08544, United States; ‡ Department of Physics and Astronomy, 14671 California State University Northridge, Northridge, California 91330, United States; § Chemistry and Nanoscience Center, National Laboratory of the Rockies, Golden, Colorado 80401, United States; ∥ Department of Electrical and Computer Engineering, University of Massachusetts, Amherst, Massachusetts 01003, United States; ⊥ Andlinger Center for Energy and the Environment, 6740 Princeton University, Princeton, New Jersey 08544, United States

**Keywords:** halide perovskites, electrochemical doping, memristors, resistive switching, endurance

## Abstract

Halide perovskites have emerged as promising materials for memristive devices. While their pronounced electrochemical reactivity and fast ionic mobility enable numerous advantages including low-voltage operation and fast switching, the same features also render perovskite-based memristors vulnerable to metallic shunts and poor endurance, limiting their practical applications. Here, we elucidate both the resistive switching and failure mechanisms in perovskite memristors with a fluorine-doped tin oxide (FTO)/methylammonium lead triiodide (MAPbI_3_)/Ag structure and demonstrate a strategy to substantially enhance device durability. As opposed to commonly invoked filamentary mechanisms, electrical, structural, and spectroscopic analyses reveal that resistive switching arises from interfacial barrier modulation by reversible Ag redox reactions that drive electrochemical doping/dedoping within the perovskite. Device failure, however, originates from metallic Ag^0^ filamentation that ultimately forms permanent conductive pathways. Introducing an ultrathin Al_2_O_3_ interlayer at the inert-electrode interface improves device endurance by more than 30-fold, exceeding 15,000 switching cycles, without compromising other performance metrics. Interfacial characterization indicates that the Al_2_O_3_ layer modifies wettability of Ag deposits, promoting planar island growth rather than through-film filamentation. These findings establish a clear link between interfacial electrochemistry, metal precipitation behavior, and memristor reliability, highlighting inert-electrode interfacial engineering as an effective pathway toward durable perovskite-based memristors.

## Graphical abstract

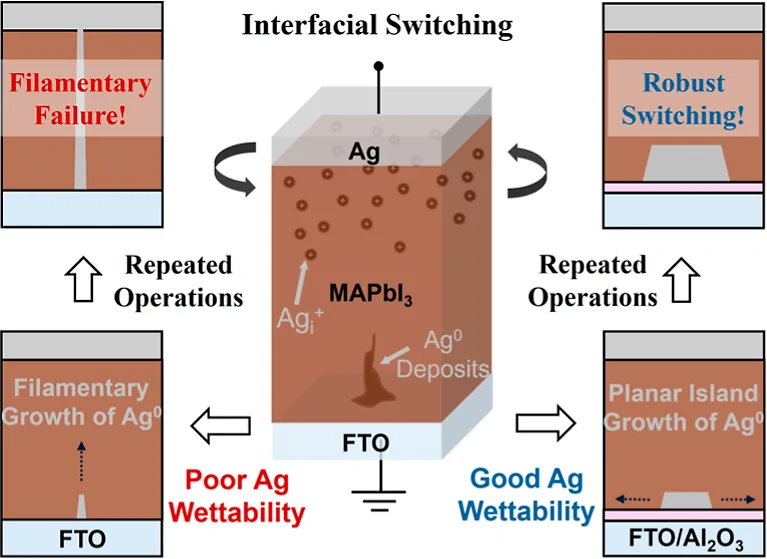

## Introduction

With the rapid advancement of artificial intelligence, the growing demand for enhanced computing capabilities at low power and high memory density has spurred intense interest in memristors as next-generation nonvolatile memory elements and artificial synapses. Memristors typically adopt a two-terminal metal–dielectric–metal architecture and offer in-memory and neuromorphic computing capabilities, high device density, and excellent scalability.^1–4^ Conventional memristors rely on binary oxide dielectrics, where voltage-driven ion redistribution enables reversible switching between distinct resistance states.^5,6^ Although the rigid lattice and strong bonding in oxides ensure long retention and robust endurance, they often require high-temperature processing and operate at relatively high voltages.^7,8^

In contrast, metal halide perovskites have recently emerged as promising alternatives for memristive applications due to their solution processability, low-voltage operation, and mechanical flexibility.^9–12^ A defining characteristic of halide perovskites is their mixed electronic–ionic conductivity: the soft metal–halide framework with structural flexibility and their relatively large lattice parameters enable rapid ion migration with low activation barriers.^13–16^ In addition, halide perovskites exhibit pronounced electrochemical reactivity with noble metal electrodes such as Au and Ag. Under modest positive bias, noble metal atoms can be oxidized to monovalent cations and injected into the perovskite lattice via interstitial sites.^17,18^ These extrinsic metal cations act as n-type dopants, increasing the conduction-band electron concentration to maintain charge neutrality. Consequently, such voltage-driven redistribution of metal ions dynamically modulates carrier density and interfacial energy barriers, giving rise to resistive switching behavior.^17,18^ Accordingly, the noble metal contact is termed the active electrode in perovskite memristive devices, as it directly participates in electrochemical processes during operation. It is typically paired with an inert electrode [e.g., fluorine-doped tin oxide (FTO)] which remains electrochemically stable within the operating voltage window. We have recently demonstrated that metal species originating from the active electrode can traverse the perovskite layer and be electroplated as metallic clusters on the inert electrode under bias.^18^

While the weak lattice bonding and high electrochemical activity of halide perovskites account for numerous advantages including low-energy fabrication, low operating voltages, and fast switching speeds, these same features also render perovskite-based memristors susceptible to excessive metal accumulation and rapid formation of permanent conductive metallic filaments.^16,19^ Such irreversible shunting under repeated cycling results in poor endurance, and the inherently stochastic nature of filament formation makes it difficult to control, which remains a major obstacle to the practical deployment of perovskite memristors.^19,20^ To address this challenge, previous efforts have largely focused on engineering the interface between the active metal electrode and the perovskite layer, such as introducing organic or oxide buffer layers, to regulate metal ion migration and thereby suppress filamentation.^21–25^ While these approaches can moderately enhance device durability from hundreds to a few thousand switching cycles, they may also inadvertently impede the essential electrochemical interactions between the active metal and the perovskite, potentially compromising key performance metrics including operating voltage and ON/OFF ratio. Consequently, an alternative strategy to suppress permanent filamentation, grounded in a detailed mechanistic understanding of both resistive switching and failure processes in perovskite memristors, is needed to achieve long-term operational robustness while preserving device functionality.

Herein, we introduce an ultrathin Al_2_O_3_ interlayer at the inert electrode/perovskite interface using atomic layer deposition (ALD) in a perovskite memristor with the structure FTO/Al_2_O_3_/methylammonium lead triiodide (MAPbI_3_)/Ag. Incorporation of the Al_2_O_3_ layer does not significantly alter the basic electrical characteristics of the reference device (without the Al_2_O_3_ interlayer), both exhibiting nonvolatile bipolar switching with an ON/OFF ratio of ∼50 and a retention time over 10^5^ s at room temperature. Remarkably, the Al_2_O_3_-incorporated memristors demonstrate a cyclic endurance exceeding 15,000 cycles, over a 30-fold improvement relative to reference devices, placing them as one of the most robust three-dimensional (3D) perovskite memristors reported to date. Further mechanistic studies reveal that the resistive switching is driven by interfacial electrochemical doping/dedoping processes and associated barrier modulation arising from redistribution of interstitial silver cations (Ag_i_^+^) and Ag^0^ species within the device, rather than relying on filamentary switching as more commonly reported. Moreover, in reference devices without the Al_2_O_3_ interlayer, repeated cyclic operations result in filamentary growth of Ag^0^ on bare FTO electrodes, ultimately causing device failure through irreversible formation of conductive pathways. In contrast, the Al_2_O_3_ coating improves Ag wettability at the inert electrode interface, promoting planar growth of more homogeneously distributed Ag precipitates and mitigating formation of penetrating filaments, which accounts for the significant endurance improvements. These results underscore interfacial engineering at the inert electrode, specifically tuning metal wettability, as a powerful approach for improving robustness of perovskite-based memristors for practical, high-performance applications.

## Results and Discussion

Figure 1a illustrates the architecture of a halide perovskite-based memristor with an FTO (ground)/Al_2_O_3_/MAPbI_3_/Ag configuration, matching to the cross-sectional scanning electron microscopy (SEM) images of the fabricated device shown in Figure 1b. The ultrathin layer of Al_2_O_3_ was coated on the FTO substrate by ALD with an estimated deposition rate of 0.11 nm per ALD cycle (Figure S1). Owing to the conformal nature of ALD, the Al_2_O_3_ layer does not noticeably modify the surface morphology of the FTO, as confirmed by atomic force microscopy (AFM) (Figure S2). Moreover, the presence of the Al_2_O_3_ interlayer does not affect the growth of the perovskite layer, as top-view SEM images reveal comparable morphology (Figure 1c,d) and grain size distribution (Figure S3) for MAPbI_3_ films deposited with and without the Al_2_O_3_ layer. In addition, X-ray diffraction (XRD) patterns (Figure S4) show nearly identical peak positions and intensities for MAPbI_3_ films on bare and Al_2_O_3_-coated FTO substrates, further confirming that the Al_2_O_3_ interlayer does not influence the structural quality or crystallinity of the perovskite film.

**Figure 1:**
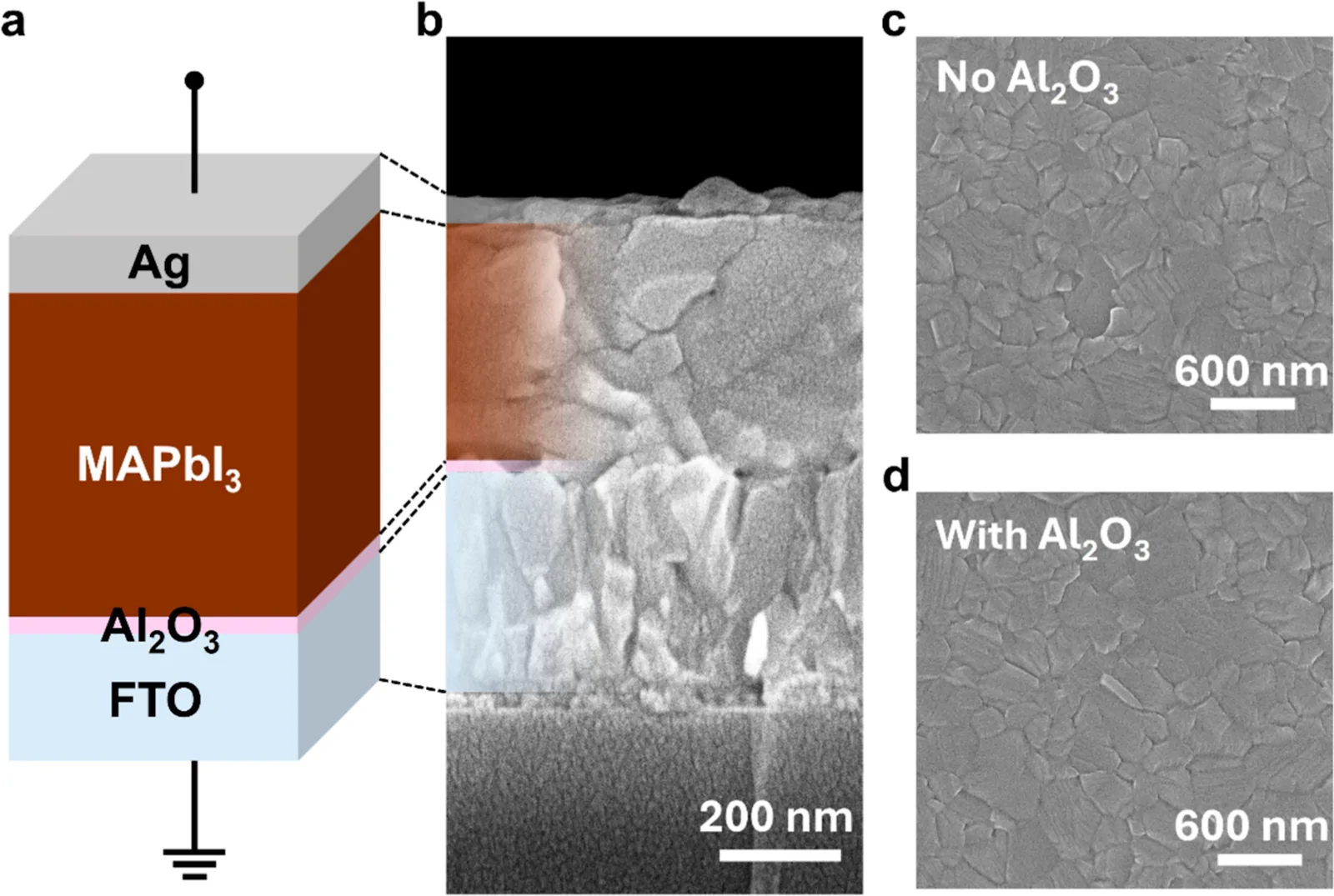
Device structure and characterization. (a) Schematic illustration of the perovskite-based memristive device. (b) Cross-sectional SEM image of the device. (c,d) SEM images of perovskite surface on (c) bare and (d) 10-ALD-cycle Al_2_O_3_-coated FTO substrates.

Prior to a detailed investigation of the electrical characteristics of the perovskite-based memristor, the thickness of the ALD-Al_2_O_3_ layer was optimized to 10 ALD cycles by evaluating the devices through cyclic switching measurements (Figure S5). Figure 2a presents the current density–voltage (*J*–*V*) characteristics of devices with and without a 10-ALD-cycle Al_2_O_3_ interlayer under a direct current (DC) voltage sweep (0 → 1.5 → 0 → −2 → 0 V). Under the positive voltage sweep, the current density increases substantially, corresponding to a SET transition from the high-resistance state (HRS) to the low-resistance state (LRS) at voltages <0.5 V. The device maintains the LRS until the applied voltage is swept to −2 V, during which the negative voltage sweep restores the current to a lower level, representing a RESET process back to its HRS. This sweep-mode operation clearly demonstrates bipolar and nonvolatile resistive switching behavior. An ON/OFF ratio of approximately 50 is achieved at a voltage of 50 mV during the voltage sweep. Notably, the *J*–*V* curves of devices with and without the Al_2_O_3_ interlayer nearly overlap, indicating almost identical quasi-DC switching behaviors. It suggests that the Al_2_O_3_ layer does not significantly affect the electrical properties and operational mechanism of the perovskite memristor as the charge carriers can easily tunnel through ultrathin Al_2_O_3_ interlayers with thicknesses on the order of 1–2 nm.^26^

**Figure 2:**
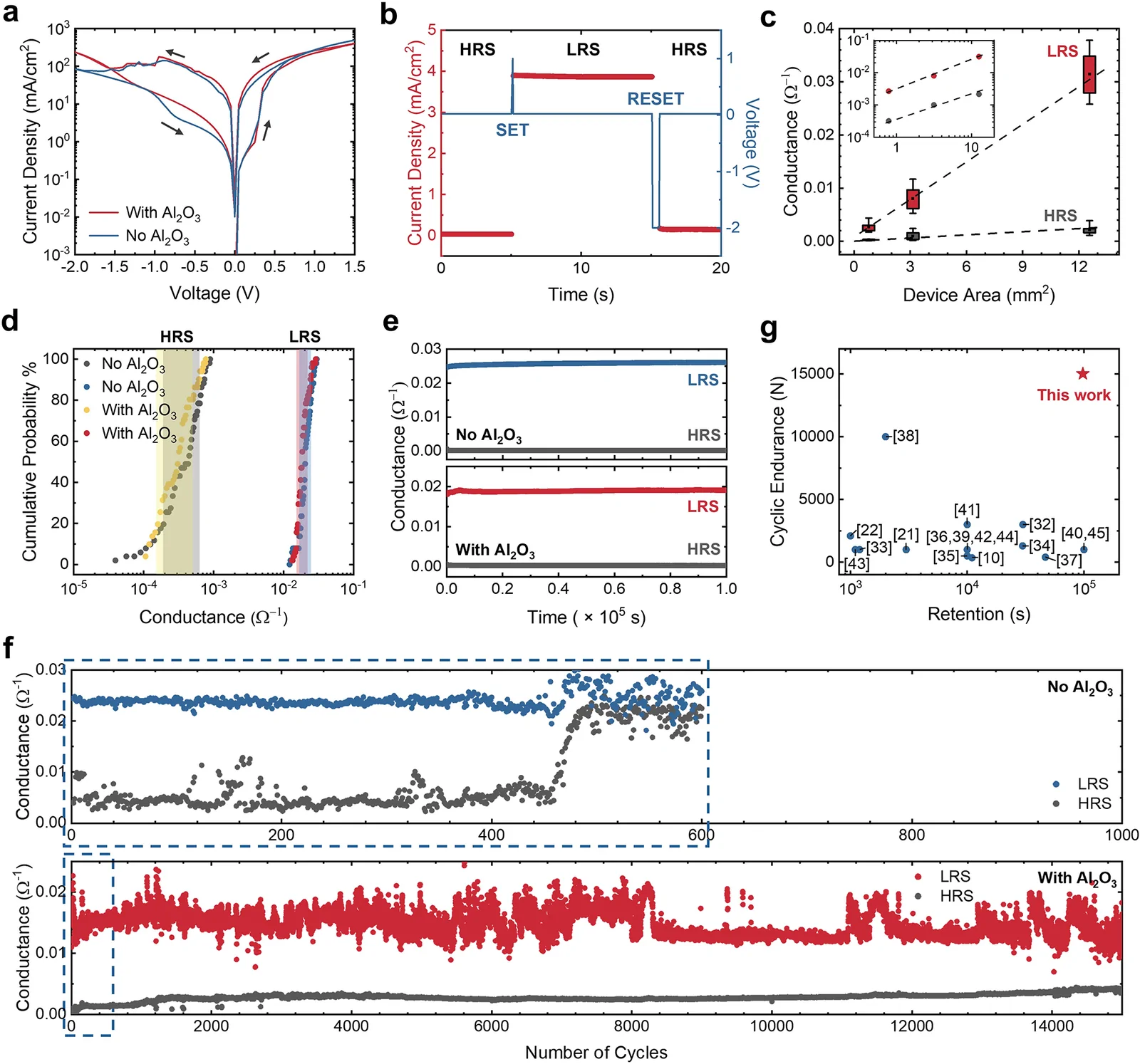
Electrical performance of perovskite-based memristive devices. (a) *J*–*V* characteristics of devices with and without a 10-ALD-cycle Al_2_O_3_ interlayer under a DC voltage sweep at a scan rate of 2250 mV/s. (b) Bipolar resistive switching driven by voltage pulses (SET: 1 V, 50 ms; RESET: −2 V, 500 ms), with the device current continuously monitored by a 20 mV readout voltage. (c) HRS/LRS conductance as a function of device area (0.79, 3.14, and 12.57 mm^2^). Error bars reflect the standard deviation across eight individual devices. The inset shows the corresponding average values on a log–log scale. (d) Cumulative distributions of HRS and LRS conductance for devices with and without the Al_2_O_3_ interlayer. Shaded regions represent the mean values and standard deviations extracted from 50 cells for each device type. (e) Room temperature HRS/LRS retention of devices with and without the Al_2_O_3_ interlayer, monitored by 20 mV readout voltages at 1 min intervals. (f) Cyclic endurance of devices with and without the Al_2_O_3_ interlayer (SET: 1 V, 50 ms; RESET: −2 V, 500 ms; read: 20 mV, 100 ms). The blue dashed boxes help visualize the substantial improvement in endurance by the Al_2_O_3_ interlayer. The relatively larger LRS variability in the Al_2_O_3_-modified device compared to the reference device is likely due to the gradual degradation of the MAPbI_3_/Ag interface during repeated switching. (g) Comparison of retention time and cyclic endurance between the Al_2_O_3_-incorporated device in this work and previously reported 3D halide perovskite-based memristors (Table S1).^10,21,22,32–45^

Pulse-mode operation of the reference perovskite memristor (without the Al_2_O_3_ interlayer) is presented in Figure 2b. The current level after the positive pulse (1 V, 50 ms) increases notably relative to the prepulse level and shows good stability during the readout period, indicating a nonvolatile transition from the HRS to the LRS. The enhanced current can be restored to its original level after a negative pulse (−2 V, 500 ms), further confirming its bipolar switching properties. Furthermore, voltage pulses (50 ms duration) with amplitudes ranging from 0.5 to 4 V were applied to the device, with each pulse delivered to a fresh cell (Figure S6). When the pulse amplitude exceeds a threshold of approximately 0.75 V at this pulse width, the device can be SET to its LRS. More importantly, the postpulse conductance increases with higher SET voltages, demonstrating analog switching behavior and multilevel storage ability that can be tuned by adjusting the pulse amplitude. The multilevel resistive switching capability of our devices is further exemplified by modulating the compliance current (*I*_cc_) during DC voltage sweeps. As shown in Figure S7a,b, at least two distinct LRS levels can be achieved by varying *I*_cc_ from 1 A to 6 mA. Additionally, a similar compliance-current-dependent LRS tunability is observed under pulse-mode operation (Figure S7c,d), where at least two LRS levels are obtained under *I*_cc_ = 1 A and 10 mA and maintained for at least 20 switching cycles, indicating the reliability of the multilevel switching behavior.

The perovskite memristors exhibit interfacial rather than filamentary resistive switching behaviors according to device area-dependent switching measurements. In filamentary switching devices, the LRS conductance is largely independent of device area due to localized current paths, whereas interfacial-type switching exhibits conductance proportional to the active area.^27^ Here, HRS and LRS conductance levels were measured for cells with areas ranging from 0.79 to 12.57 mm^2^ before and after application of a SET pulse (1 V, 50 ms). As shown in Figure 2c, both HRS and LRS conductance values scale linearly with the device area while maintaining a nearly constant ON/OFF ratio (inset of Figure 2c), indicative of interfacial-type switching. To further probe the role of the top electrode, we replaced Ag with Au, a less electrochemically active metal, and performed both sweep- and pulse-mode measurements on FTO/MAPbI_3_/Au devices. Under DC voltage sweeps with an extended voltage range (−4 V ↔ 4 V), these devices only exhibit diode characteristics with weak hysteresis, rather than bipolar resistive switching (Figure S8a,b). Additionally, a SET pulse (1 V, 50 ms) applied on the Au-based device does not produce a measurable change in conductance (Figure S8c,d), whereas the same pulse induces a substantial conductance increase in Ag-based devices (Figure 2b). The Au-based devices remain unswitchable even when the pulse amplitude is increased to 4 V. These results indicate that the FTO/MAPbI_3_/Au devices do not exhibit bipolar switching under the bias conditions applied in this study, likely due to insufficient doping arising from slower kinetics of Au/MAPbI_3_ interactions (i.e., higher voltage thresholds to oxidize Au and higher migration energy for gold cations).^18^ Moreover, the absence of nonvolatile switching properties in the Au-based devices, even under aggressive biasing conditions, strongly indicates that the migration of intrinsic iodide species is not the primary origin of resistive switching and, by itself, is insufficient to induce switching without the involvement of an electrochemically active metal electrode. Collectively, these findings support a resistive switching mechanism governed by voltage-induced interfacial electrochemical interactions between Ag and the perovskite (see more discussion in Note S1).

The resistive switching mechanism of perovskite memristors was further explored by replotting the *J*–*V* characteristics on a double-logarithmic scale (Figure S9a). The HRS conduction can be divided into three distinct regimes. At low bias (*V* < 0.2 V), the double-log plot shows a linear dependence with a slope of ∼1.18, reflecting ohmic conduction dominated by the drift of intrinsic carriers in the perovskite. This ohmic behavior remains valid with the presence of the ultrathin Al_2_O_3_ interlayer as direct charge tunneling through alumina interlayers with similar thicknesses (∼1–2 nm) has been reported to be appreciable even at low bias.^26^ Upon increasing the bias (0.2 V < *V* < 0.6 V), the current exhibits a pronounced superlinear increase with a slope of ∼5.06, characteristic of rapid trap filling by carriers injected from the electrodes. In our system, this process may be further assisted by the incorporation of Ag_i_^+^. As Ag begins to be oxidized at intermediate voltages, a substantial population of Ag_i_^+^ dopants is injected into the perovskite layer, progressively narrowing the interfacial depletion region and thereby contributing to the concomitant rapid increase in current. Moreover, the absence of an abrupt current surge implies a nonfilamentary switching. At higher voltages (0.6 V < *V* < 1.5 V), the slope decreases to ∼1.56, reflecting a transition to quasi-ohmic transport. This behavior is attributed to direct tunneling-dominated charge injection through a sufficiently narrow depletion region at a metal/heavily doped semiconductor interface. Specifically, substantial local Ag_i_^+^ donor accumulation near the perovskite/Ag interface significantly reduces the depletion width, so that direct charge tunneling through the interfacial barrier with quasi-ohmic behavior becomes increasingly favorable and eventually dominates over conventional thermionic emission in this regime (Figure S9b).^28^ This quasi-ohmic feature persists at LRS during the reverse voltage sweep back to 0 V, with a slightly reduced slope of ∼1.32. For comparison, the *J*–*V* curve of a device which was shorted during the positive voltage sweep is included in Figure S9c. An abrupt current jump occurs at 1.75 V, after which the device is irreversibly stuck at a low-resistance state. Replotting the data on a double-log scale (Figure S9d) reveals that the “LRS” of the failed device during the reverse scan features a linear dependence with a slope of ∼1.08, indicating ohmic conduction. This behavior confirms that device failure originates from the formation of a permanent conductive filament. Additionally, the observation that device failure only occurs during the positive voltage sweep implies that the SET condition may play a major role in metallic filamentation and device failure.

Furthermore, the interfacial switching mechanism is consistent with the *J*–*V* characteristics obtained under sweep-mode operations with different compliance currents (i.e., *I*_cc_ = 6, 10, 20, and 1000 mA). As shown in Figure S7a,b, the LRS conductance does not show a strong dependence on compliance current once *I*_cc_ exceeds 10 mA: the *J*–*V* curves nearly overlap, and the LRS conductance approaches saturation. This behavior is in clear contrast to the filamentary-type perovskite memristors where the LRS conductance typically scales strongly with *I*_cc_, as the applied compliance current intrinsically influences the thickness of conductive filaments.^29,30^ In our devices, unlike conductive filaments that can continuously thicken under higher compliance currents, the dopant concentration near the MAPbI_3_/Ag interface likely possesses an upper limit and can become saturated under cyclic sweeping. Nevertheless, when a sufficiently low compliance current (*I*_cc_ = 6 mA) is applied, a reduced LRS conductance is observed. We attribute this behavior to incomplete interfacial doping caused by the limited applied voltage under the low-current condition.

The functionality and performance of our perovskite memristors were then evaluated based on their switching uniformity, resistance retention, and cyclic endurance. First, the device-to-device uniformity was evaluated across 50 individual cells for each device type, with each cell SET by a 1 V pulse with a duration of 50 ms. The cumulative distributions of HRS and LRS conductance level for both configurations are presented in Figure 2d. The average HRS and LRS conductance values of the control devices are (4.15 ± 2.23) × 10^–4^ Ω^–1^ and (2.10 ± 0.39) × 10^–2^ Ω^–1^, respectively, while those of the devices with Al_2_O_3_ are (3.31 ± 1.78) × 10^–4^ Ω^–1^ and (1.90 ± 0.34) × 10^–2^ Ω^–1^. The distribution profiles show clear distinction between the high and low resistance states, corresponding to average ON/OFF ratios of 50.5 and 57.3 for devices with and without the Al_2_O_3_ layer, respectively, consistent with values obtained from sweep-mode measurements. While these ON/OFF ratios are relatively modest compared to those reported for other 3D iodide perovskite-based memristors (typically ranged from 10^2^ to 10^3^),^31^ we note that such high ON/OFF ratios are generally achieved in filamentary-type devices with miniaturized dimensions, as the HRS resistance increases with decreasing device area while the LRS remains area-independent. In contrast, both the LRS and HRS conductance levels in our interfacial devices scale proportionally with device area (Figure 2c), thereby limiting the achievable ON/OFF ratio even upon device scaling. The larger device-to-device variation observed in the HRS likely arises from the high sensitivity of barrier-limited transport to local interfacial and material inhomogeneities, whereas the LRS is stabilized by high-concentration Ag-induced doping that homogenizes charge transport and reduces variability. Notably, the conductance distributions of both resistance states for devices with and without the Al_2_O_3_ layer largely overlap (Figure 2d), implying again that the ultrathin Al_2_O_3_ interlayer does not significantly affect the switching behavior of the perovskite memristor.

To assess the reliability of the perovskite memristors, pulse retention tests were performed on devices both with and without the Al_2_O_3_ interlayer. As shown in Figure 2e, devices with and without the Al_2_O_3_ interlayer maintain stable ON/OFF ratios of approximately 27 and 37, respectively, for durations exceeding 10^5^ s, with minimum current fluctuation in both resistance states, demonstrating excellent retention performance. Furthermore, cyclic switching endurance was characterized to evaluate the durability of the perovskite memristors with and without the Al_2_O_3_ interlayer. As presented in Figure 2f, the reference device exhibits failure after approximately 460 cycles, at which point the HRS becomes irreversibly stuck at an elevated conductance level. This RESET failure is likely caused by the formation of a permanent metallic filament bridging the electrodes, arising from the repeated incorporation of Ag cations and their subsequent reduction to neutral Ag^0^ within the perovskite layer during cycles of operations. In stark contrast, the device with the Al_2_O_3_ interlayer remains functional for more than 15,000 switching cycles, representing an endurance enhancement more than 30× relative to our reference device and highlighting their exceptional operational robustness among recently reported 3D perovskite memristors, which typically operate for a few thousand cycles (Figure 2g).^10,21,22,32–45^ The presence of the Al_2_O_3_ layer at the inert electrode interface is therefore inferred to suppress the formation of penetrating Ag filaments, accounting for the dramatic improvement in switching endurance.

To directly verify the suppression of filamentary Ag growth on the Al_2_O_3_-coated FTO electrodes, we performed SEM characterization of the bottom electrode interfaces of a failed reference device after extended cyclic switching (failed after approximately 300 cycles) and an Al_2_O_3_-modified device after reliably switching for 2000 cycles. The bottom electrode interfaces were exposed by mechanically removing the Ag electrode using polyimide tape, followed by washing off the perovskite layer with *N*,*N*-dimethylformamide (DMF). For these measurements, a 1 nm lithium fluoride (LiF) layer was thermally evaporated between the perovskite and the Ag top electrode to facilitate Ag delamination (see Methods). The introduction of the LiF layer does not significantly affect the bipolar switching behavior of the devices, except for leading to a slightly higher turn-on voltage (Figure S10). As shown in Figure S11, SEM images reveal the presence of bright precipitates at the bottom interface in both device configurations, which were identified as Ag^0^ clusters by energy dispersive X-ray (EDX) mapping, confirming Ag movement and redistribution during cyclic switching. However, distinct differences in Ag morphology are observed. In the failed device, Ag precipitates feature whisker-like structures on bare FTO, which suggests filamentary growth and may contribute to the conductive pathways responsible for device failure. In contrast, Ag on Al_2_O_3_-coated FTO exhibits improved wettability, manifesting as planar island-like growth. The absence of metallic whiskers at the Al_2_O_3_-coated interface effectively mitigates detrimental filamentation, thereby enabling the exceptionally improved device endurance.

To accelerate Ag precipitation and further elucidate the influence of the Al_2_O_3_ layer on the spatial distribution of Ag species, potentiostatic biasing experiments were performed on devices with and without the Al_2_O_3_ interlayer at applied voltages ranging from 0.3 to 0.8 V for 5 min, with each bias condition tested on a fresh cell. The resulting current density–time (*J*–*t*) traces under potentiostatic bias are shown in Figure S12, exhibiting comparable current levels for both device configurations, consistent with efficient carrier tunneling through the Al_2_O_3_ layer. This similarity excludes current-induced Joule heating as the origin of any observed differences in Ag redistribution between the two device types.

Following biasing, the Ag electrodes and the perovskite layers were sequentially removed to expose the bottom interface. Subsequent SEM–EDX analysis reveals Ag^0^ precipitates on the underlying FTO substrate in both device configurations (Figures 3a,b, S13 and S14), similar to the observations made after cyclic operations (Figure S11). The SEM images of the FTO interfaces from unbiased cells with and without the Al_2_O_3_ coating are provided in Figure S13 for reference. Similar metal plating behavior has been reported in ITO/MAPbI_3_/Au devices, where Au atoms from the top contact are oxidized under anodic bias, migrate through the perovskite bulk as gold interstitial cations (Au_i_^+^), and subsequently redeposit as Au^0^ clusters at the bottom cathodic interface.^18^ Prior studies further indicate that Ag can undergo a similar anodic oxidation process, readily forming monovalent cations at interstitial sites within the perovskite lattice, analogous to Au_i_^+^.^17,46^ Accordingly, the observed Ag^0^ precipitates are most likely produced through an analogous electrolytic plating mechanism.

**Figure 3:**
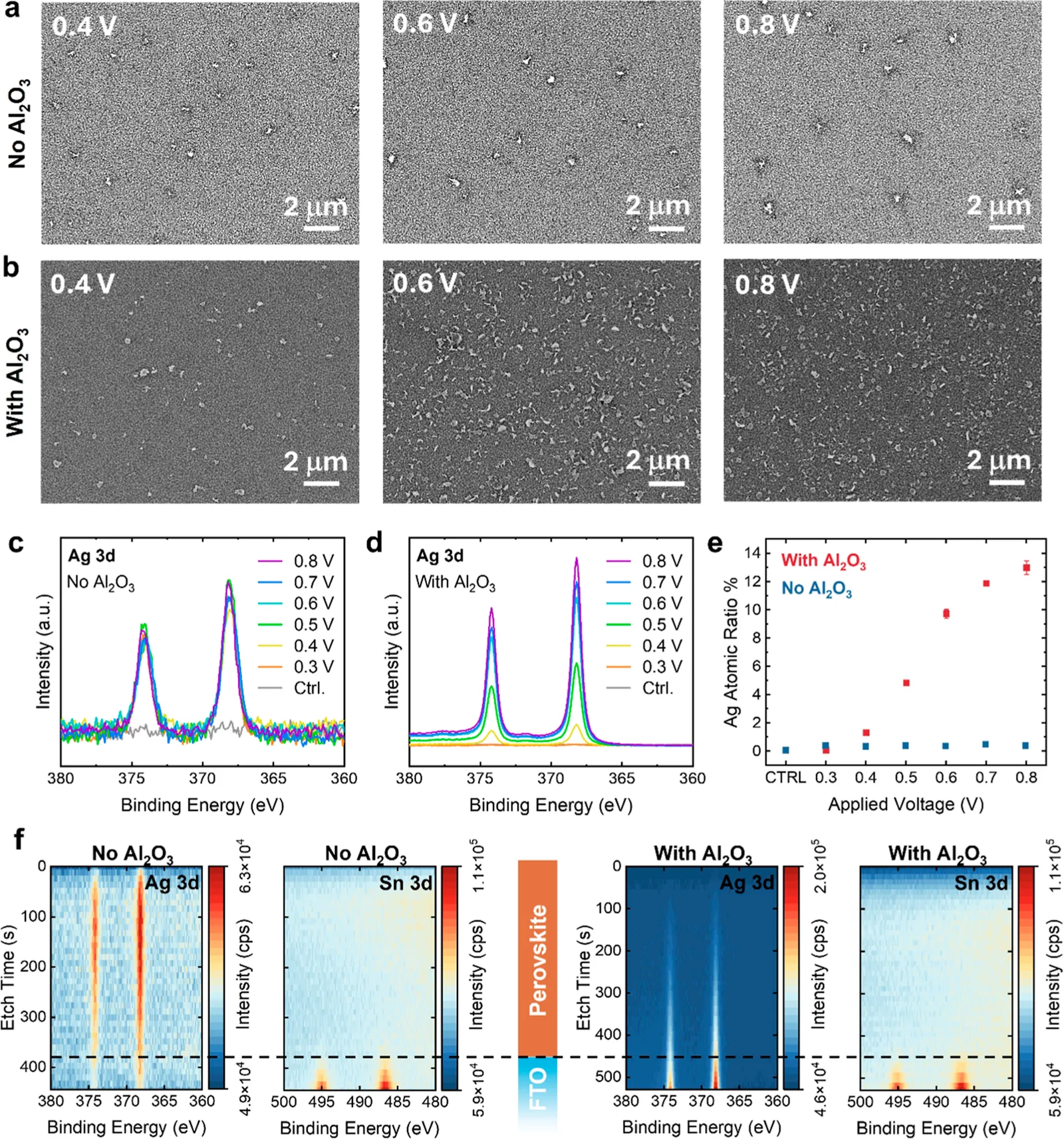
Influence of the Al_2_O_3_ interlayer on Ag plating morphology and distribution. (a,b) SEM images of (a) bare and (b) Al_2_O_3_-coated FTO substrates after perovskite removal, following potentiostatic biasing at different voltages. (c–e) XPS measurements at the exposed cathodic interfaces after perovskite removal, following 5 min biasing at indicated voltages: Ag 3d spectra of devices with (c) bare and (d) Al_2_O_3_-coated FTO cathodes; (e) Ag atomic percentage as a function of applied bias. The Ag atomic percentage is defined as the ratio of Ag atoms to the total number of atoms detected within the XPS measurement volume. Error bars in (e) reflect the standard deviation of Ag atomic percentages measured by XPS. (f) XPS depth profiles of Ag 3d and Sn 3d signals from devices with and without the Al_2_O_3_ layer after Ag delamination, following 5 min of biasing at 0.7 V.

Consistent with the results observed from prolonged cycling, the presence of the Al_2_O_3_ coating substantially alters the morphology of the plated Ag. As shown in Figures 3a and S13, Ag precipitates formed on bare FTO substrates are more sparsely distributed and exhibit pointed features, whereas those on Al_2_O_3_-coated substrates display higher surface coverage and predominately planar growth, as highlighted by a zoom-in image in Figure S14. This contrast becomes increasingly evident at higher voltages (Figures 3b and S13). Furthermore, the SEM images suggest that the overall amount of Ag deposited on bare FTO substrates remains relatively insensitive to the applied voltage, whereas both the size and areal density of Ag islands on Al_2_O_3_-coated substrates increase monotonically with bias. This trend is further corroborated by X-ray photoelectron spectroscopy (XPS) analysis of the exposed bottom interfaces. The Ag 3d signal intensity for bare FTO samples remains nearly constant across all bias conditions (Figure 3c), corresponding to an approximately constant Ag atomic ratio of 0.4% (Figure 3e), In contrast, Al_2_O_3_-coated samples show a pronounced bias-dependent increase in Ag 3d peak intensity (Figure 3d), with the Ag atomic ratio rising sharply from ∼0.1% to ∼13% as the voltage increases from 0.3 to 0.8 V (Figure 3e). Collectively, these observations suggest better Ag wettability on Al_2_O_3_ relative to FTO: Ag^0^ preferentially nucleates and spreads laterally at the Al_2_O_3_ interface whereas on bare FTO it may tend to grow vertically, a morphology that is more prone to forming penetrating, detrimental filamentation.

Moreover, XPS depth profiling was employed to further examine Ag redistribution within the perovskite layer on devices with and without the Al_2_O_3_ interlayer following biasing at 0.7 V for 5 min. To prevent driving Ag into the perovskite underlayer by ion etching, Ag top electrodes were mechanically delaminated prior to XPS measurements. This procedure results in clean removal, as no Ag signal is detected either at the surface or throughout the bulk of an unbiased control sample (Figures S15 and S16). As presented in Figure 3f, the sample without the Al_2_O_3_ interlayer exhibits a relatively uniform Ag distribution throughout the perovskite thickness, with detectable Ag signals at both the top and bottom interfaces (Figure S16), indicating a potential in forming penetrating Ag filaments under this bias condition. On the other hand, the device incorporating the Al_2_O_3_ interlayer displays an asymmetric Ag depth profile, with Ag signals concentrated near the bottom cathodic interface (Figure 3f). Notably, no Ag signal is detected at the top perovskite interface in the Al_2_O_3_-based sample (Figure S16), suggesting a suppressed formation of penetrating filaments and correspondingly reduced likelihood of device failure. These results align with the observed morphology of Ag precipitates at the exposed cathode interface (Figure 3a–e), collectively indicating better wettability of Ag on the Al_2_O_3_ coating.

The wettability and growth mode of solid precipitates on a substrate are largely governed by the surface energy of the substrate. Hence, contact angle measurements were performed to compare the surface energies of bare and Al_2_O_3_-coated FTO using two common probe liquids, diiodomethane (DIM) and deionized water (H_2_O). As shown in Figure S17, a pronounced difference is observed when water is in contact with the substrates: it beads up on bare FTO with a contact angle of 58.9°, whereas it spreads more readily on the Al_2_O_3_-coated surface, exhibiting a smaller contact angle of 29.4°, indicative of a higher surface energy for the Al_2_O_3_ coating. As detailed in Note S2, the absolute surface energies of bare and Al_2_O_3_-coated FTO are calculated to be 53.9 and 69.1 mJ/m^2^ respectively based on Fowkes surface energy theory.^47^ Indeed, coating the FTO with Al_2_O_3_ increases the surface energy, which might result in a higher interfacial energy between the perovskite and the bottom substrate. Thus, as Ag deposits, it might tend to replace this high-energy interface by favoring in-plane, lateral growth, providing a plausible rationalization for the enhanced Ag wettability observed on Al_2_O_3_. We note that directly quantifying the interfacial energies between the perovskite and the substrates would be highly informative; however, such measurements are challenging due to the lack of suitable polar probe liquids that do not chemically degrade the perovskite layer. Similarly, directly measuring the interfacial energy between Ag and the substrates is also difficult, as it would, in principle, require molten Ag. Therefore, future systematic studies combining advanced interfacial characterization and theoretical modeling are encouraged to fully elucidate the interplay among interfacial energies (between the perovskite, metal precipitates, and substrates), metal deposition morphology, and device reliability, thereby facilitating the development of more effective strategies for reliability enhancement.

We also acknowledge that interfacial energy might not be the only contributing factor to the improved Ag wetting on Al_2_O_3_-coated FTO. It is also possible that the Al_2_O_3_ interlayer contributes to a more uniform electric potential across the FTO surface, thereby promoting more uniform Ag deposition. Specifically, bare FTO may contain sparse, highly electrochemically active sites that preferentially nucleate Ag under bias. Once Ag precipitates form at such reactive sites, the local electric field can be further enhanced, driving progressively more localized growth that ultimately cascades into through-film filaments and device failure,^48^ manifesting as the sparse, pointed Ag morphologies observed in Figures 3a and S11a. It is also consistent with the voltage-independent amount of plated Ag on bare FTO after potentiostatic biasing, as evidenced by both SEM and XPS (Figure 3a,e). By contrast, the Al_2_O_3_ coating likely passivates these reactive sites, resulting in more numerous and uniformly distributed nucleation events. This homogenizes postnucleation and mitigates electrical potential inhomogeneity, contributing to the spatially dense, planar Ag morphology observed in Figures 3b and S11b, which enables nonfilamentary growth and robust device performance.

Collectively, the observations presented in this study provide a comprehensive understanding of the resistive switching and failure mechanisms in perovskite memristors, as well as elucidate how the incorporation of an ultrathin Al_2_O_3_ interlayer at the inert electrode interface significantly enhances device robustness. To begin with, area-dependent switching behavior (Figure 2c), together with quasi-ohmic conduction at LRS (Figure S9a), point to an interfacial-type resistive switching mechanism likely associated with voltage-induced redox reactions between Ag and the perovskite. Specifically, resistive switching in these devices is proposed to be driven by electrochemical doping/dedoping processes and corresponding interfacial barrier modulation arising from redistribution of Ag_i_^+^/Ag^0^ species within the perovskite layer (Figure 4a). Initially, the device resides in a HRS due to the low intrinsic carrier concentration of the perovskite film and the presence of a Schottky barrier at the perovskite/Ag interface, which suppresses charge transport (Figure 4b, initial HRS). Upon applying a positive bias to the Ag electrode, Ag atoms are oxidized and injected into the perovskite lattice as interstitial cations (Ag_i_^+^), which have been reported to act as n-type dopants in halide perovskites.^17^ They increase the free carrier concentration and substantially narrow the depletion region width at the MAPbI_3_/Ag Schottky interface, facilitating carrier tunneling and leading to a pronounced enhancement in device conductivity, thereby switching the device to a LRS (Figure 4b, LRS). This mechanism accounts for the SET process during positive voltage sweeps (Figure 1a) and following the positive voltage pulses (Figure 2b). Varying the voltage pulse amplitude modulates the rate of electrochemical reactions and the extent of Ag_i_^+^ dopant incorporation, thus enabling multilevel switching as shown in Figure S6. Meanwhile, this SET voltage also drives Ag_i_^+^ migration toward the FTO electrode, where the cathodic environment reduces Ag_i_^+^ reaching the interface to Ag^0^, giving rise to the Ag precipitates observed in Figure S11. Our potentiostatic biasing experiments emulate this process and accelerate the evolution of the Ag^0^ deposits.

**Figure 4:**
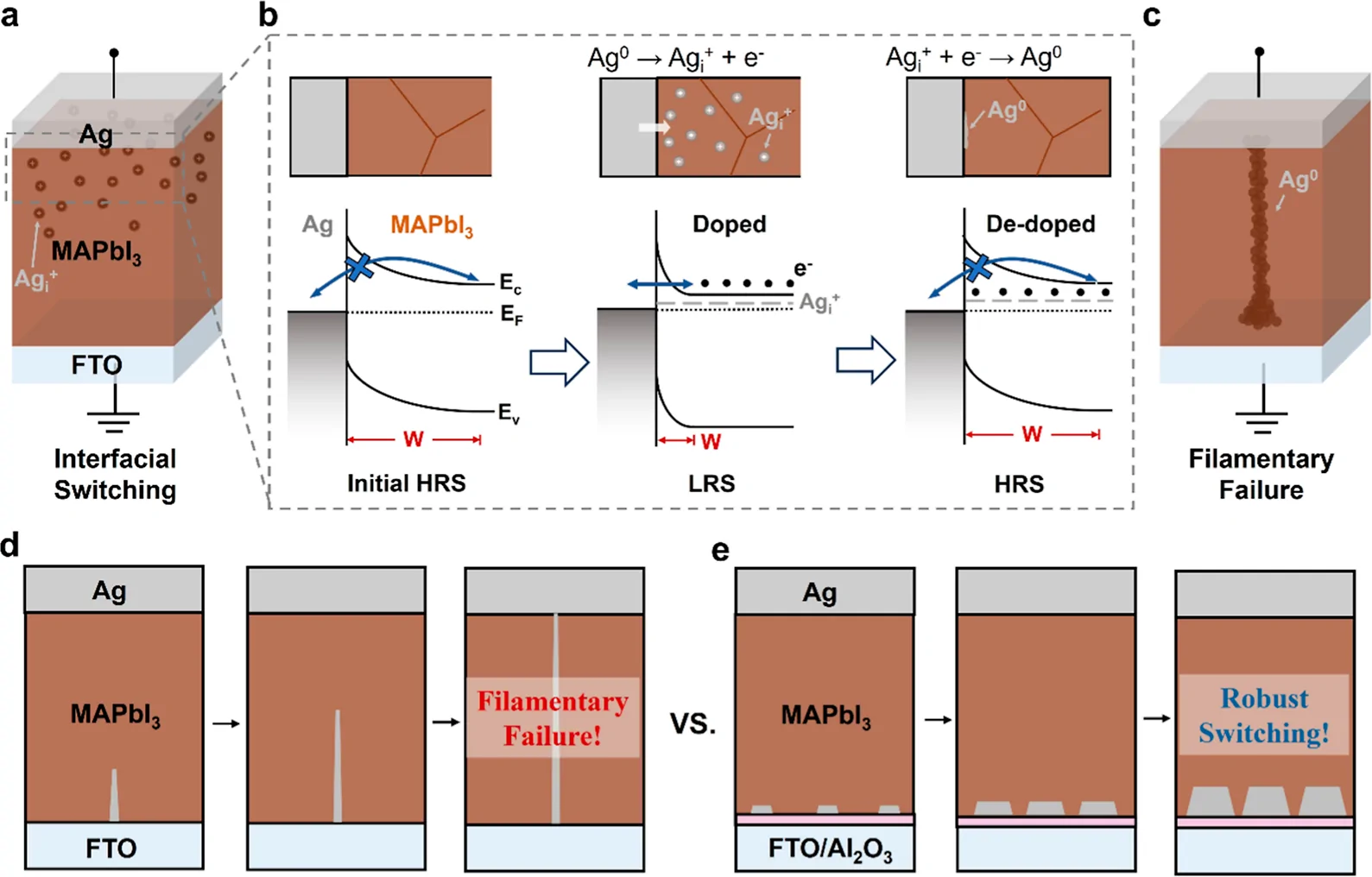
Proposed resistive switching and failure mechanisms in perovskite memristors. (a) Interfacial-type resistive switching driven by electrochemical reactions at the perovskite/Ag interface. (b) Schematics illustration of Ag_i_^+^/Ag^0^ species redistribution near the perovskite/Ag interface at different resistance states, together with corresponding qualitative energy diagrams highlighting Ag-induced doping/dedoping and interfacial barrier modulation as the switching mechanism. (c) Filamentary failure arising from the irreversible formation of a conductive Ag^0^ filament. (d,e) Proposed growth modes of precipitated Ag^0^ on (d) bare and (e) Al_2_O_3_-coated FTO substrates after repeated switching cycles, exhibiting filamentary and planar island growth, respectively. Full-device schematics illustrating both the switching and failure mechanisms can also be found in Figure S18.

The RESET process occurs when a negative bias (either a voltage sweep or pulse) is applied to the Ag contact. The resulting cathodic condition at the perovskite/Ag interface reduces Ag_i_^+^ cations to metallic Ag^0^ species, which likely exit the perovskite lattice and plate back onto the Ag contact under the applied field, effectively dedoping the perovskite bulk. Consequently, the depletion width at the Schottky interface is restored, returning the device to its high-resistance state (Figure 4b, HRS). While the RESET voltage renders the bottom FTO electrode anodic and could, in principle, reoxidize the plated Ag^0^, such recovery is limited,^18^ resulting in gradual Ag accumulation during cyclic operation.

Under prolonged cycling, repeated accumulation of metallic Ag^0^ may evolve into filamentary growth. Once a conductive pathway ultimately bridges the two electrodes, the perovskite layer becomes permanently shunted, resulting in device failure (Figure 4c). This mechanism likely explains the failure mode of devices without the Al_2_O_3_ interlayer after cyclic operations or during the positive voltage sweep (Figure 4d). In contrast, the presence of the Al_2_O_3_ coating promotes lateral growth of Ag precipitates at the bottom interface, suppressing the formation of penetrating filaments and thereby reducing the likelihood of device failure (Figure 4e). As a result, the Al_2_O_3_-incorporated devices exhibit substantially enhanced cyclic endurance.

Introducing a barrier layer, such as Al_2_O_3_, at the perovskite top interface is a widely adopted strategy in perovskite solar cells to suppress ion migration, including oxidized metal species sourcing from the top electrode, and thereby enhance device longevity by preventing metallic shunt formation.^49–51^ However, applying similar top-interface modification strategies to perovskite memristive devices is not ideal, as their switching behavior relies on electrochemical reactions between the active metal electrode and the perovskite. Having a physical barrier to metal ion transport could adversely affect key device metrics, including switching speed and operating voltages. On the contrary, engineering the inert electrode interface provides an effective means to suppress filamentary growth of precipitated metal and mitigate detrimental shunting without compromising the electrochemically driven switching functionality at the active metal/perovskite interface. In fact, similar strategies are widely utilized in solid-state batteries (SSBs) to improve device robustness. During battery charging, lithium ions undergo comparable redox processes where they transport through the electrolyte and are electrolytically plated as metallic Li^0^ on the negatively biased electrode (typically lithium metal). Upon repeated cycles of operations, the deposited Li^0^ can evolve into penetrating dendrites, ultimately causing short circuits and device failure. In response, extensive SSB research has focused on modifying the lithium–metal-anode/electrolyte interface via protective coatings and properly controlling interphase formation to suppress the initiation of Li dendrite formation,^52–54^ such as hollow carbon nanospheres to isolate Li metal deposition,^55^ atomic-layer-deposition of alumina to enhance interfacial wettability,^56^ and polymer coatings to homogenize current distribution^57^ or mechanically suppressing dendrite propagation.^58^ The similarities in failure mechanisms between perovskite memristors and SSBs not only underscore their shared basis in electrochemistry but also highlight interfacial engineering at the inert electrode as a powerful yet overlooked strategy to suppress detrimental filamentation and improve endurance in perovskite memristive devices. Future systematic investigations, such as the exploration of different inert electrode coating layers in combination with various active and inert electrodes, would be valuable for evaluating the broader applicability of this approach, gaining deeper insight into the failure mechanisms, and identifying more effective strategies for reliability enhancement.

## Conclusion

In summary, this work provides a mechanistic understanding of both the resistive switching and failure behaviors in perovskite-based memristors and introduces an effective strategy to significantly enhance device robustness. We demonstrate that resistive switching stems from an interfacial-type mechanism driven by voltage-induced electrochemical doping/dedoping of the perovskite via reversible redistribution of Ag_i_^+^/Ag^0^ species, distinguishing it from commonly reported filamentary switching and vacancy doping processes. This process modulates the carrier concentration and the depletion width at the perovskite/Ag Schottky interface, enabling nonvolatile bipolar switching without the formation of localized conductive filaments during normal operation. On the other hand, device failure is traced to filamentary growth of metallic Ag^0^ under repeated cycling that eventually bridges the electrodes, leading to permanent shunting. By inserting an ultrathin Al_2_O_3_ interlayer at the inert-electrode interface, we effectively suppress this failure pathway and achieve a more than 30-fold improvement in cyclic endurance (>15,000 switching cycles). Structural and spectroscopic analyses reveal that this enhancement originates from altered Ag wettability on Al_2_O_3_ compared to bare FTO, resulting in larger densities and planar growth of Ag precipitates rather than sparsely distributed, vertically penetrating filaments. This change in growth mode mitigates filament formation while preserving the electrochemical interactions at the active Ag/perovskite interface that are essential for memristive functionality, underscoring the critical role of inert-electrode interfacial engineering in improving device reliability. More broadly, the parallels between failure mechanisms in memristors and other electrochemical systems highlight interfacial wettability control as an effective strategy for achieving operational durability in ionically active electronic devices.

## Methods

### Materials and Sample Fabrication

Lead iodide (PbI_2_, 99.99% trace metal basis) and methylammonium iodide (MAI, >99.99%) were purchased from TCI and GreatCell Solar, respectively. *N*,*N*-dimethylformamide (DMF, anhydrous, 99.8%), chlorobenzene (CB, anhydrous, 99.8%), and lithium fluoride (LiF, anhydrous, ≥99.99% trace metal basis) were purchased from Sigma-Aldrich. Prepatterned fluorine-doped tin oxide (FTO) substrates (3 × 3 cm^2^, TEC15) were purchased from Xin Yan Technology, Ltd. All materials and solvents were used as received.

The patterned FTO substrates were sequentially cleaned by ultrasonication in detergent, deionized water, acetone, and isopropanol for 10 min each, followed by a 10 min O_2_ plasma treatment. After cleaning, Al_2_O_3_ were deposited onto the FTO substrates using a Gemstar XT thermal atomic layer deposition (ALD) system (Arradiance), with trimethylaluminum and water serving as the metal and oxidant precursors, respectively. The substrate temperature was maintained at 125 °C during the ALD process, and the Al_2_O_3_ thickness was tuned by adjusting the number of ALD cycles. The Al_2_O_3_-coated substrates were treated with O_2_ plasma for 10 min again prior to perovskite deposition. Methylammonium lead triiodide (MAPbI_3_) precursor solutions were prepared inside a N_2_-filled glovebox by dissolving PbI_2_ and MAI at a concentration of 1.2 M with a 1:1 molar ratio in DMF. Inside a N_2_-filled glovebox, the MAPbI_3_ films were subsequently deposited by spin coating the prepared precursor solution using a two-step protocol: 1000 rpm for 10 s with a ramping rate of 1000 rpm/s, followed by 6000 rpm for 30 s with a ramping rate of 2000 rpm/s. 100 μL of CB was dropped onto the substrate 25 s before the end of the entire spin-coating program as an antisolvent. The samples were immediately annealed on a hot plate at 100 °C for 30 min. Subsequently, 100 nm of Ag was thermally evaporated under vacuum. The Ag top electrodes were patterned using a shadow mask, defining devices with an active area of ∼0.1 cm^2^ (4 × 2.5 mm^2^).

To expose the FTO/MAPbI_3_ interface for characterizing and quantifying Ag precipitation after extended cyclic switching or potentiostatic biasing, we need to first physically delaminate the Ag electrode using polyimide tape and subsequently remove the perovskite layer with DMF. However, Ag is difficult to peel from MAPbI_3_ when the two layers are in direct contact, likely due to stronger adhesion between Ag and MAPbI_3_. Therefore, an ultrathin LiF interlayer (1 nm) was thermally evaporated prior to Ag deposition to facilitate Ag delamination.

### Electrical Characterizations

All electrical measurements were performed in a N_2_-filled environment in the dark at room temperature using a Keithley 2602B sourcemeter controlled by SCPI Matlab scripts. All electrical measurements were performed under a compliance current of 1 A unless otherwise specified. Direct current (DC) voltage sweeps were performed at a scan rate of 2250 mV/s (Figures 2a, S7,S8 and S10). For the pulse-mode operation (Figure 2b), a positive (1 V, 50 ms) and a negative (−2 V, 500 ms) voltage pulse were sent to SET and RESET the device sequentially, whereas a constant readout voltage (*V*_read_) of 20 mV was used to monitor the resistance before and after the voltage pulses. Multilevel switching was achieved by applying positive voltage pulses (50 ms duration) with different amplitudes (i.e., 0.5, 0.75, 1, 2, and 4 V) to the devices, with each pulse delivered to a fresh cell (Figure S6). During retention tests (Figure 2e), a 1 V SET pulse with a duration of 50 ms was first applied to switch the device to its LRS. This was followed by a sequence of nondestructive read pulses (20 mV, 100 ms) applied at 1 min interval to monitor the LRS over time. Subsequently, the device was returned to the HRS using a −2 V RESET pulse with a duration of 0.5 s, after which the same readout sequence was used to track the HRS stability. During endurance measurements (Figures 2f and S5), the devices were repeatedly switched between the two resistance states using SET pulses (1 V, 50 ms) and RESET pulses (−2 V, 500 ms), with current readout voltages (20 mV, 100 ms) in between to monitor the resistances states throughout successive cycles.

### Material Characterizations

Scanning electron microscopy (SEM) images and energy-dispersive X-ray spectroscopy (EDX) were measured by an FEI Verios 460 XHR SEM. Samples were coated with 3 nm of iridium before SEM measurements. The atomic force microscopy (AFM) images were obtained using a VEECO Dimension 3100 atomic force microscope at tapping mode. X-ray diffraction (XRD) was performed using a Bruker D8 Discovery X-ray diffractometer equipped with a copper X-ray source (wavelength 1.54 Å) and a scintillation detector (0.1 mm divergence slits). Ellipsometry measurements were conducted using a Woollam M-2000 ellipsometer to measure the Al_2_O_3_ thickness on Si substrates. X-ray spectroscopy (XPS) measurements were performed by a Thermo-Scientific Kα X-ray Photoemission Spectrometer operating at a base pressure of <1 × 10^–7^ mbar and using an Al anode at a power of 72 W. The XPS depth profiles were obtained by ion etching using a 2000 eV ion energy. Contact angle measurements were performed using a Ramé-hart contact angle goniometer at room temperature under ambient conditions.

## Supplementary Material



## Data Availability

The data that support the findings of this study are available from the corresponding author upon reasonable request.

## Notes

Estimation of ALD deposition rate; morphology comparison between bare and Al_2_O_3_-coated FTO substrates; Statistical grain size analysis and XRD patterns of perovskite films on bare and Al_2_O_3_-coated FTO substrates; optimization of the ALD-Al_2_O_3_ thickness; multilevel resistive switching of FTO/MAPbI_3_/Ag devices by tuning the amplitude of the SET voltage pulses; electrical characteristics of Ag-based devices under different compliance currents; electrical characteristics of Au-based devices under aggressive biasing conditions; *J*–*V* characteristics of the FTO/MAPbI_3_/Ag device on a double logarithmic scale, compared with that of a failed device; SEM–EDX characterizations of Ag precipitates on bare and Al_2_O_3_-coated FTO substrates after extended cyclic operations and long-term biasing; *J*–*t* characteristics of devices with and without the Al_2_O_3_ interlayer during potentiostatic biasing at various voltages; XPS depth profiles of an unbiased FTO/Al_2_O_3_/MAPbI_3_/LiF/Ag device; contact angle measurements on bare and Al_2_O_3_-coated FTO substrates and the calculation of substrate surface energies; full-device schematics for switching and failure mechanism; summary of cyclic endurance and retention time of recently reported 3D perovskite-based memristors; discussion on the role of iodide species in resistive switching (PDF)

## #

Querrey Simpson Institute for Bioelectronics, Northwestern University, Evanston, IL 60208, USA
