# Supplementary material for: From Filamentary Failure to Durable Halide Perovskite Memristors
Source: ACS Nano. 2026 Aug 5;20(32):22562–73. doi: 10.1021/acsnano.6c04620 (PMC13492475; doi:10.1021/acsnano.6c04620)
Supplement: Supplementary file 1 [file nn6c04620_si_001.pdf]

Supporting Information

From Filamentary Failure to Durable Halide Perovskite Memristors

Tuo Hu,[a] *Nathalie Tuazon*,[b] *Zhaojian Xu*,[a†] *Sunney Gao*,[a] *Jisu Hong*,[a] *Hussein Badran*,[a] *Ross A. Kerner*,[c] *Qiangfei Xia*,[d*] and *Barry P. Rand*[a,e*]

[a] Department of Electrical and Computer Engineering, Princeton University, Princeton, NJ 08544, USA

[b] Department of Physics and Astronomy, California State University Northridge, Northridge, CA 91330, USA

[c] Chemistry and Nanoscience Center, National Laboratory of the Rockies, Golden, CO 80401, USA

[d] Department of Electrical and Computer Engineering, University of Massachusetts, Amherst, MA 01003, USA

[e] Andlinger Center for Energy and the Environment, Princeton University, Princeton, NJ 08544, USA

[†] Current Affiliation: Querrey Simpson Institute for Bioelectronics, Northwestern University, Evanston, IL 60208, USA

[*] Correspondence: qxia@umass.edu (Q.X.) and brand@princeton.edu (B.P.R.)

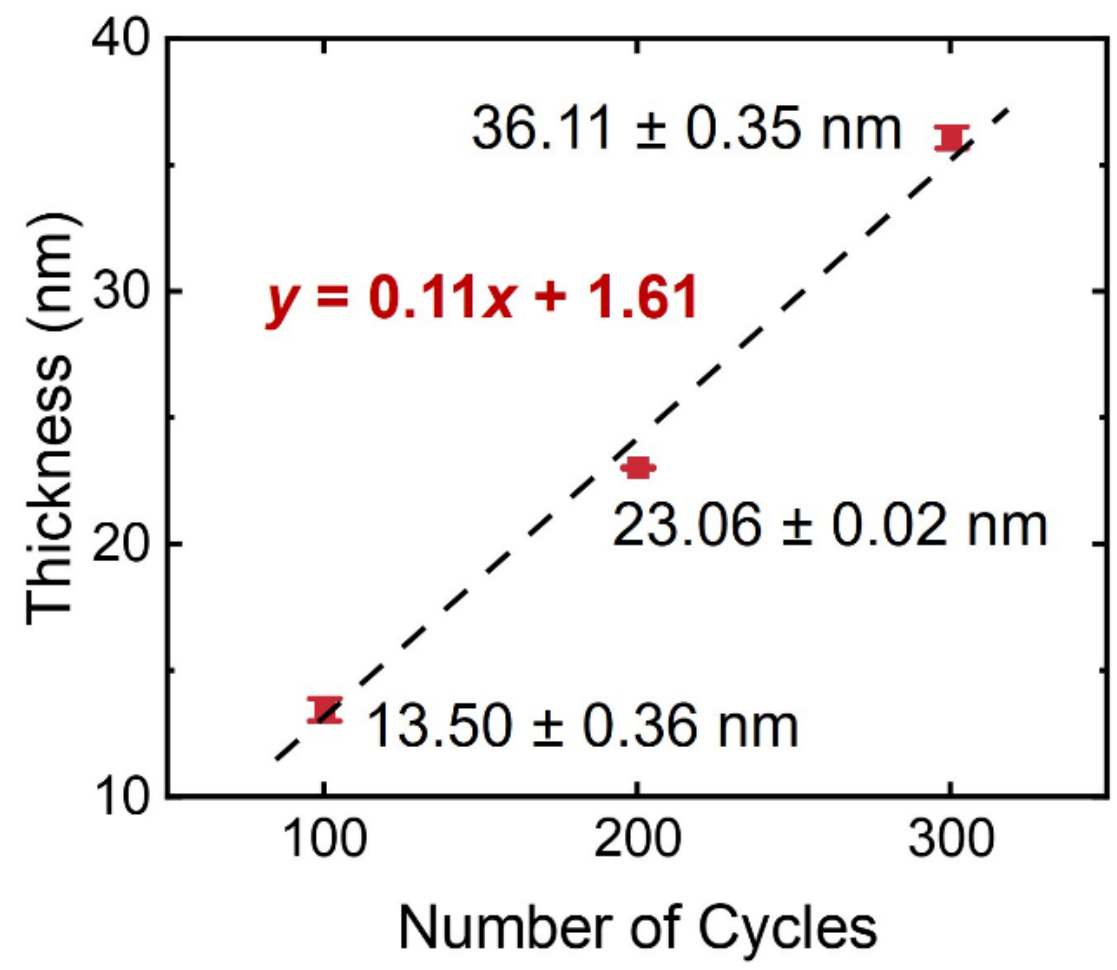

Figure S1. Al_2O_3 thickness as a function of the number of ALD cycles measured by ellipsometry. Error bars reflect the standard deviation of thickness values obtained from three independent samples. The Al_2O_3 thickness increases linearly with the number of ALD cycles, corresponding to a growth rate of approximately 0.11 nm per cycle. Note that the nonzero y-intercept of 1.61 nm implies uncertainty in accurate thickness determination at low ALD cycle numbers. Accordingly, we avoid explicitly assigning precise Al_2O_3 thickness values in this work.

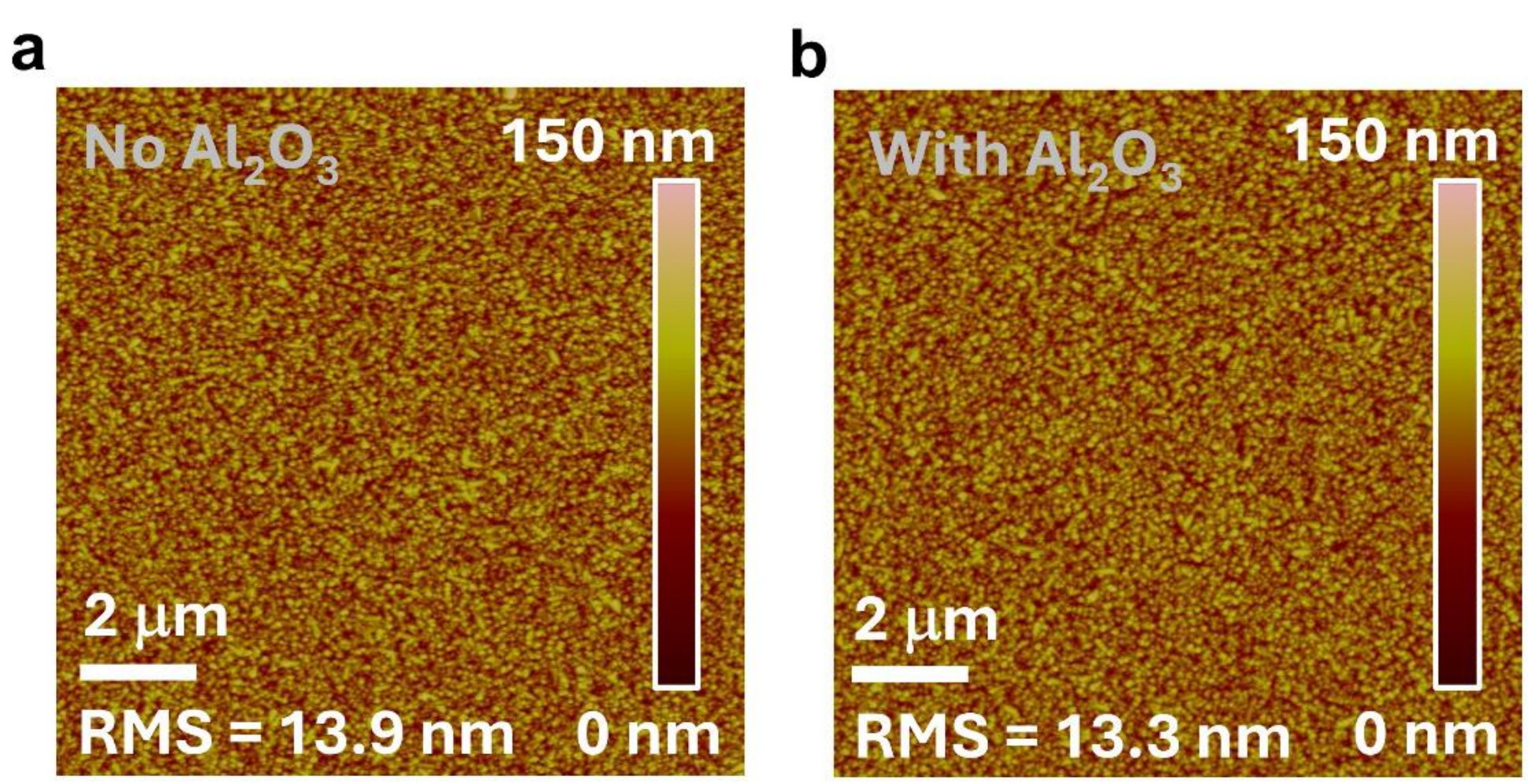

Figure S2. AFM images of (a) bare and (b) 10-ALD-cycle Al_2O_3-coated FTO substrates.

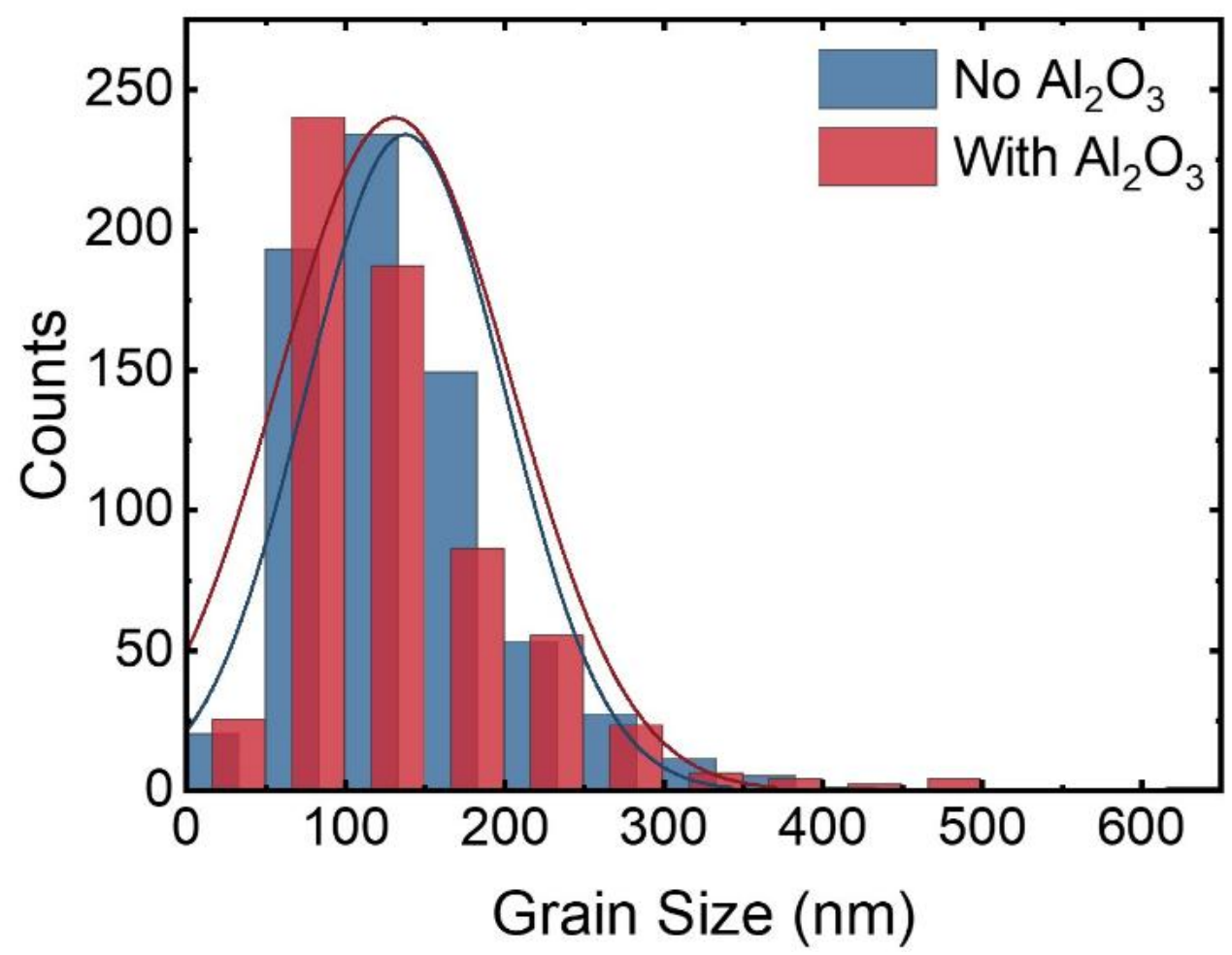

Figure S3. Statistical grain size analysis of perovskite films deposited on bare and Al_2O_3-coated FTO substrates. The average grain sizes are 137.6 ± 62.8 nm and 130.7 ± 73.6 nm for films on bare and Al_2O_3-coated FTO, respectively.

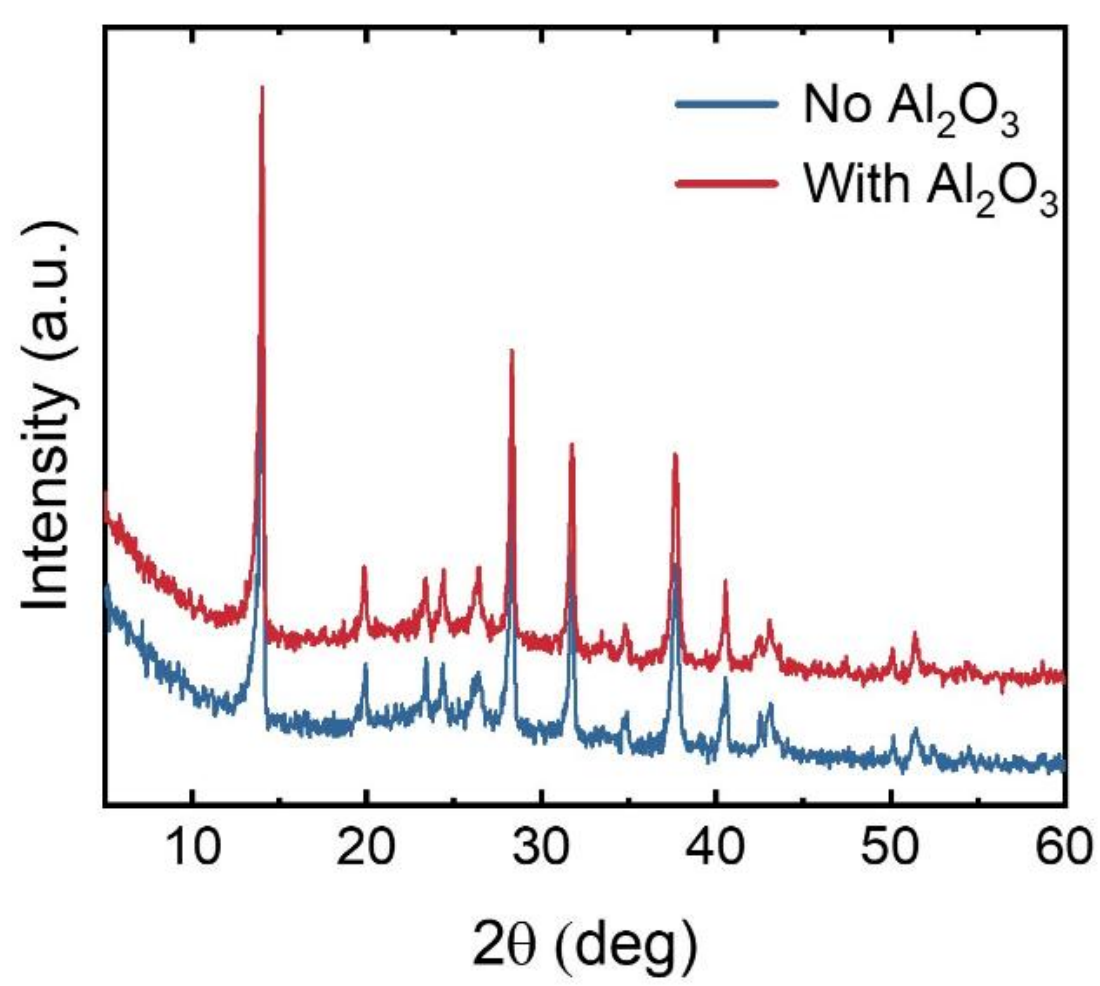

Figure S4. X-ray diffraction (XRD) patterns of perovskite films deposited on bare and Al_2O_3-coated FTO substrates.

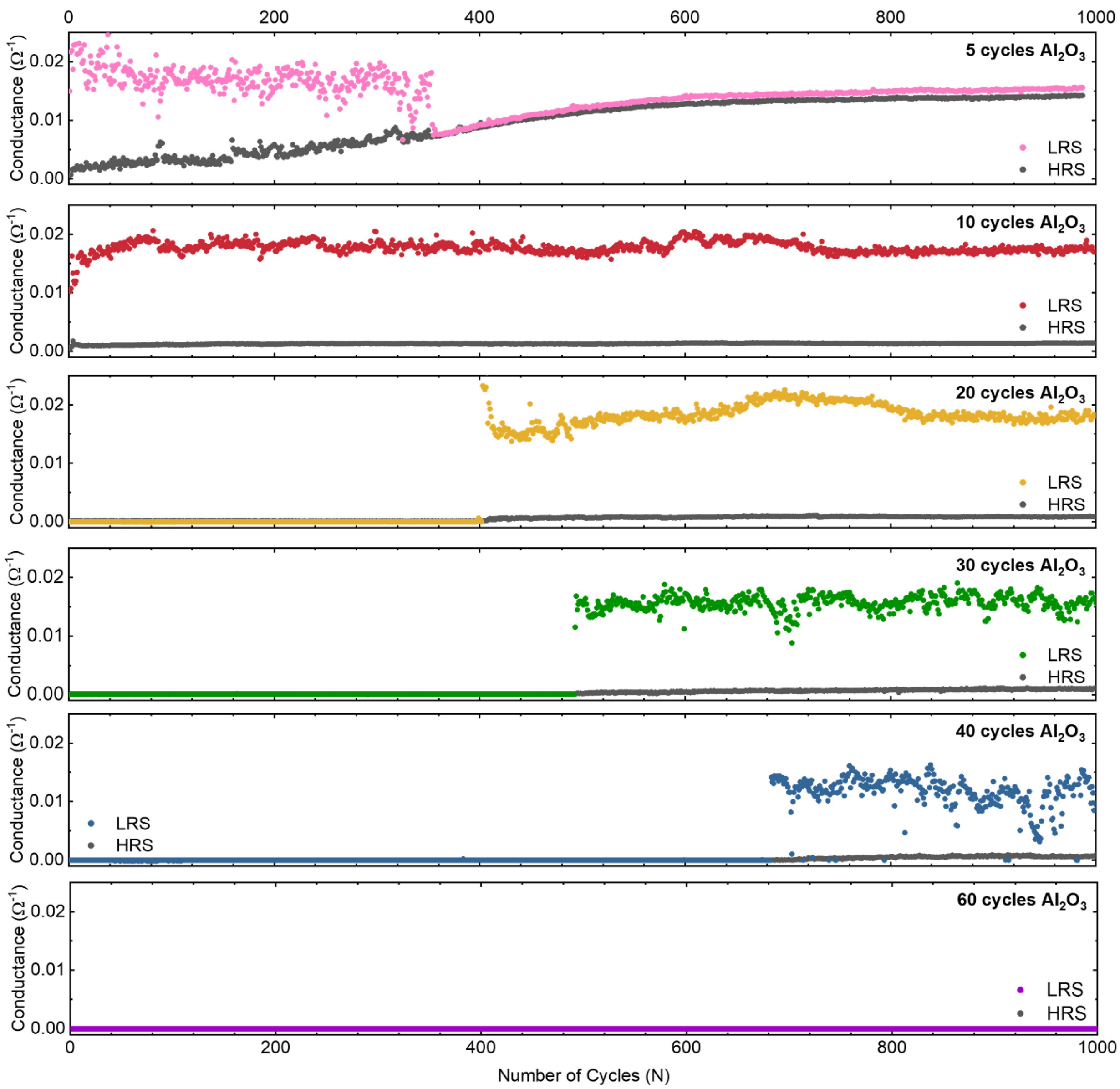

Figure S5. Optimization of the Al_2O_3 interlayer thickness (i.e., the number of ALD cycles) based on the cyclic endurance performance of the corresponding memristive device (SET: 1 V, 50 ms; RESET: -2 V, 500 ms; Read: 20 mV, 100 ms). Devices with a 5-cycle Al_2O_3 layer show no noticeable improvement compared to devices without the interlayer (Figure 2f). Increasing the Al_2O_3 thickness beyond 10 ALD cycles leads to delayed switching which is not ideal for practical operation. Consequently, 10 ALD cycles are identified as the optimal thickness for the Al_2O_3 interlayer.

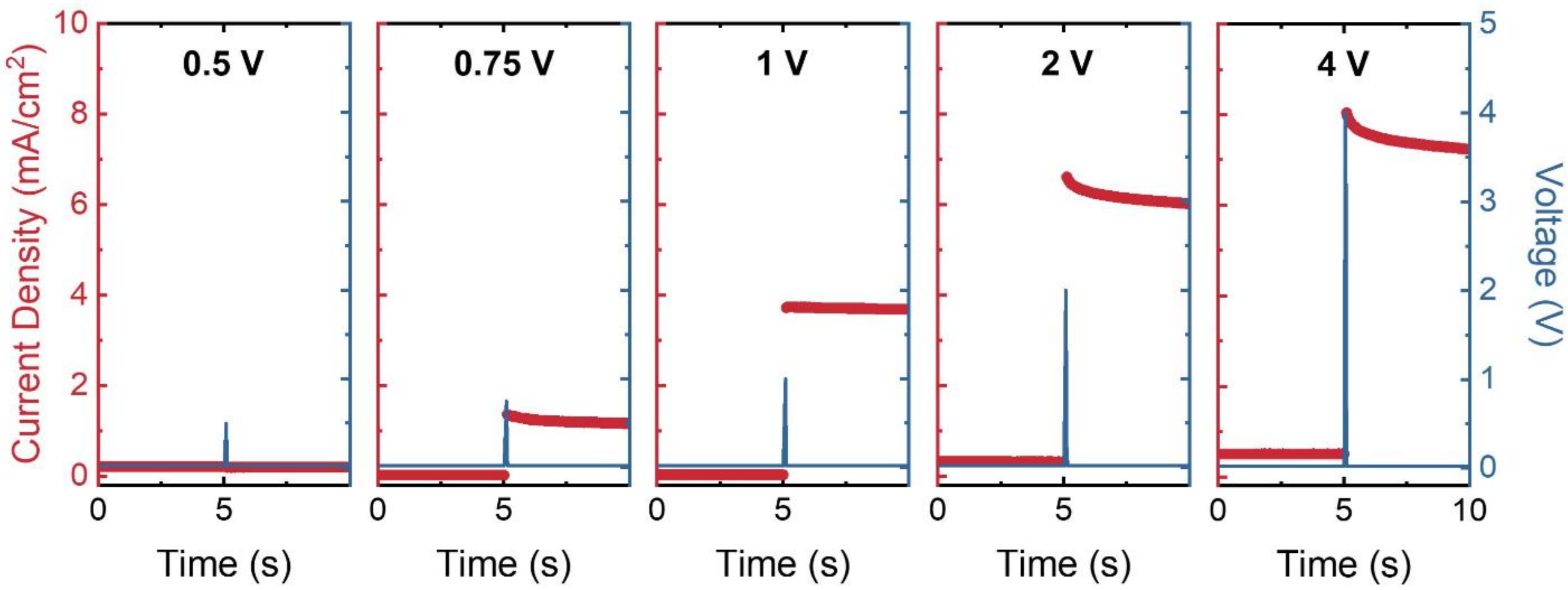

Figure S6. Multilevel resistive switching achieved by tuning the amplitude of the SET voltage pulse. Each pulse (50 ms duration) was applied to a fresh device. The current was continuously monitored using a 20 mV readout voltage.

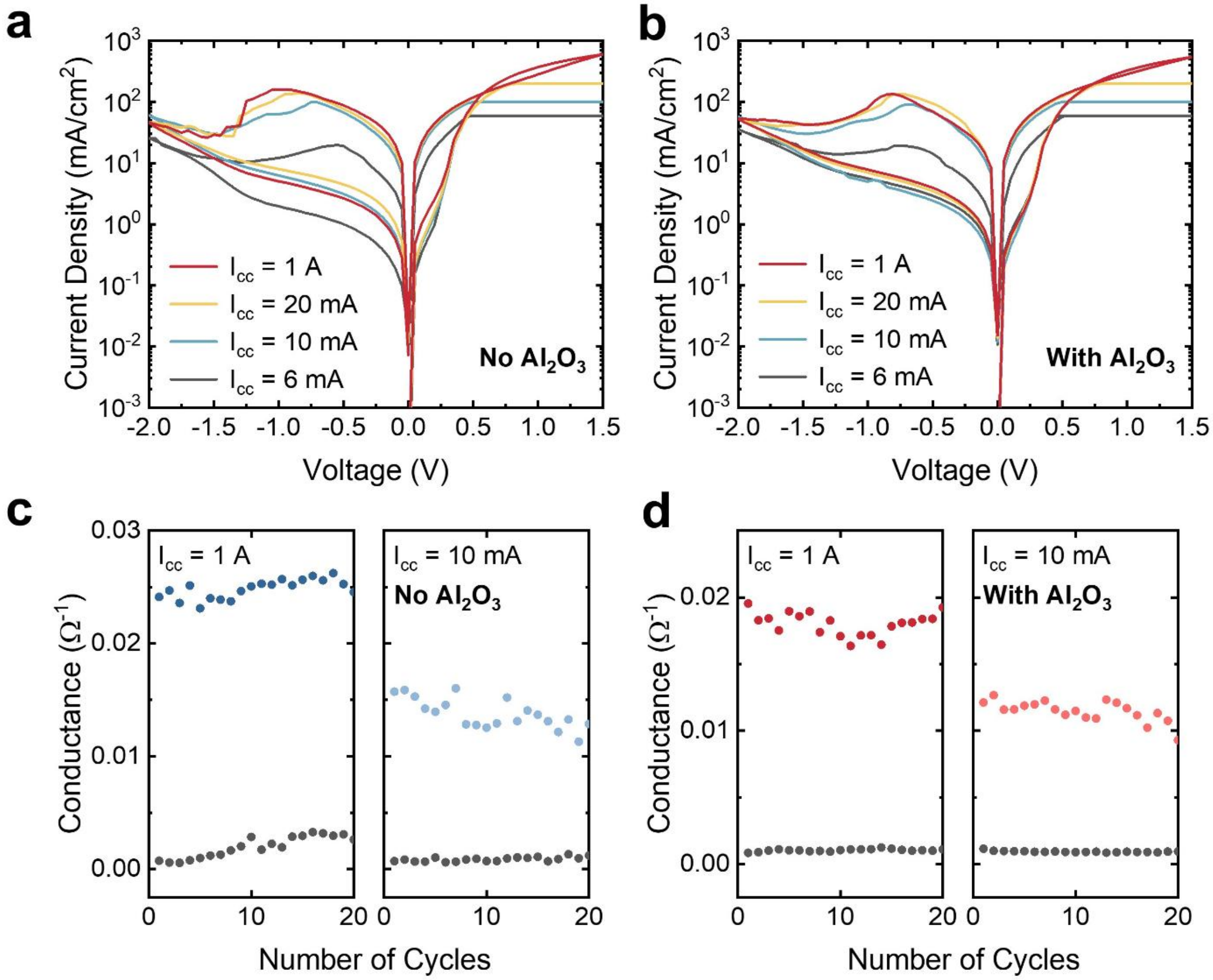

Figure S7. Electrical characteristics of perovskite-based memristors under different compliance currents (I_{cc}). Current density–voltage (*J–V*) characteristics of the (a) reference and (b) Al_2O_3-modified devices under direct current (DC) voltage sweeps with I_{cc} = 6, 10, 20, or 1000 mA. Bipolar resistive switching of the (c) reference and (d) Al_2O_3-modified devices driven by voltage pulses (SET: 1 V, 50 ms; RESET: -2 V, 500 ms) under I_{cc} = 10 or 1000 mA, with the resistance state monitored by a 20 mV readout voltage.

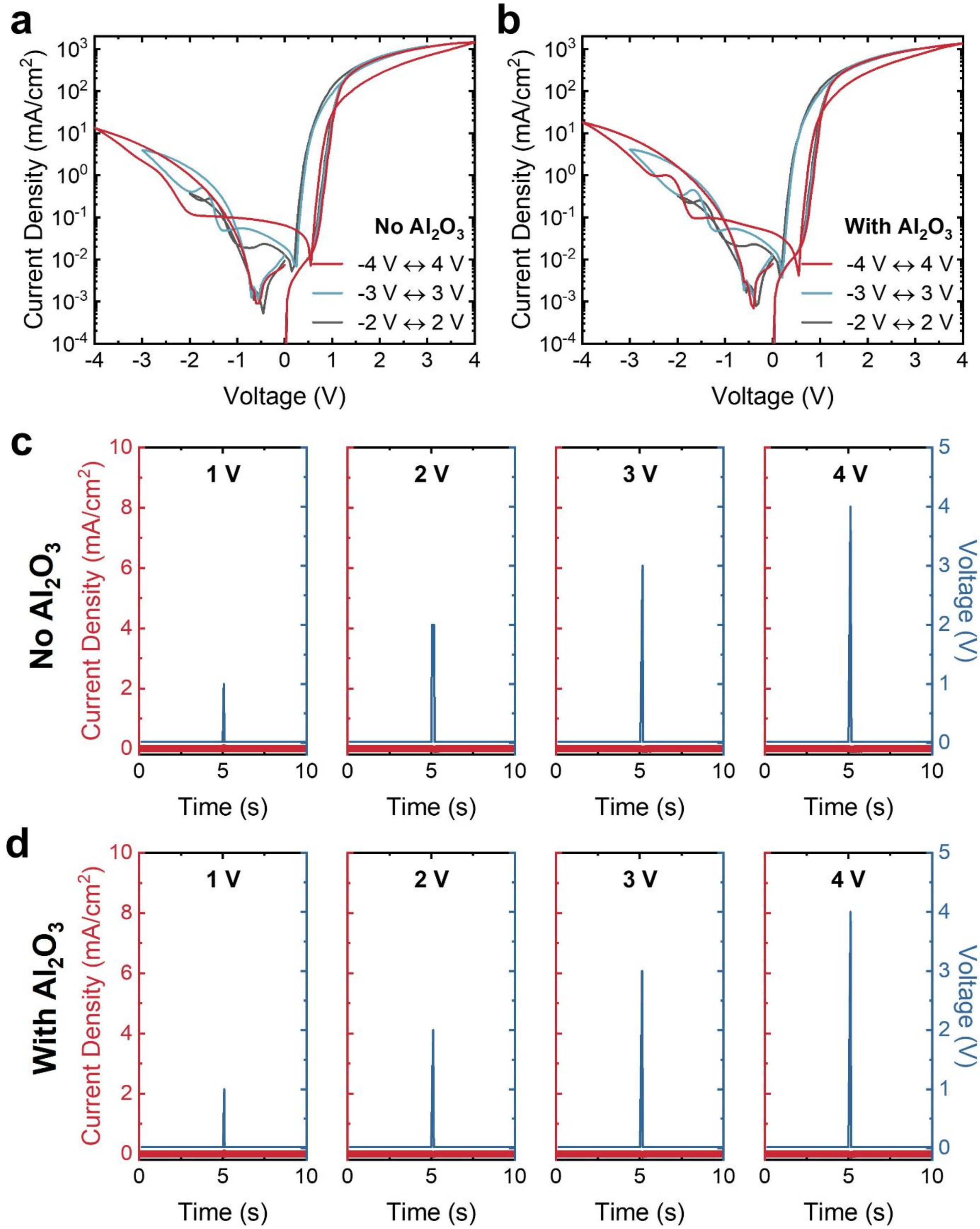

Figure S8. Electrical characteristics of FTO/$MAPbI_3$/Au devices. *J–V* characteristics of (a) the no-Al_2O_3 reference and (b) Al_2O_3-modified devices measured under DC voltage sweeps with various sweep ranges (i.e., -2 V ↔ 2 V, -3 V ↔ 3 V, and -4 V ↔ 4 V). Current response to SET voltage

pulses (50 ms) with different amplitudes (i.e., 1, 2, 3, and 4 V). The current was continuously monitored using a 20 mV readout voltage.

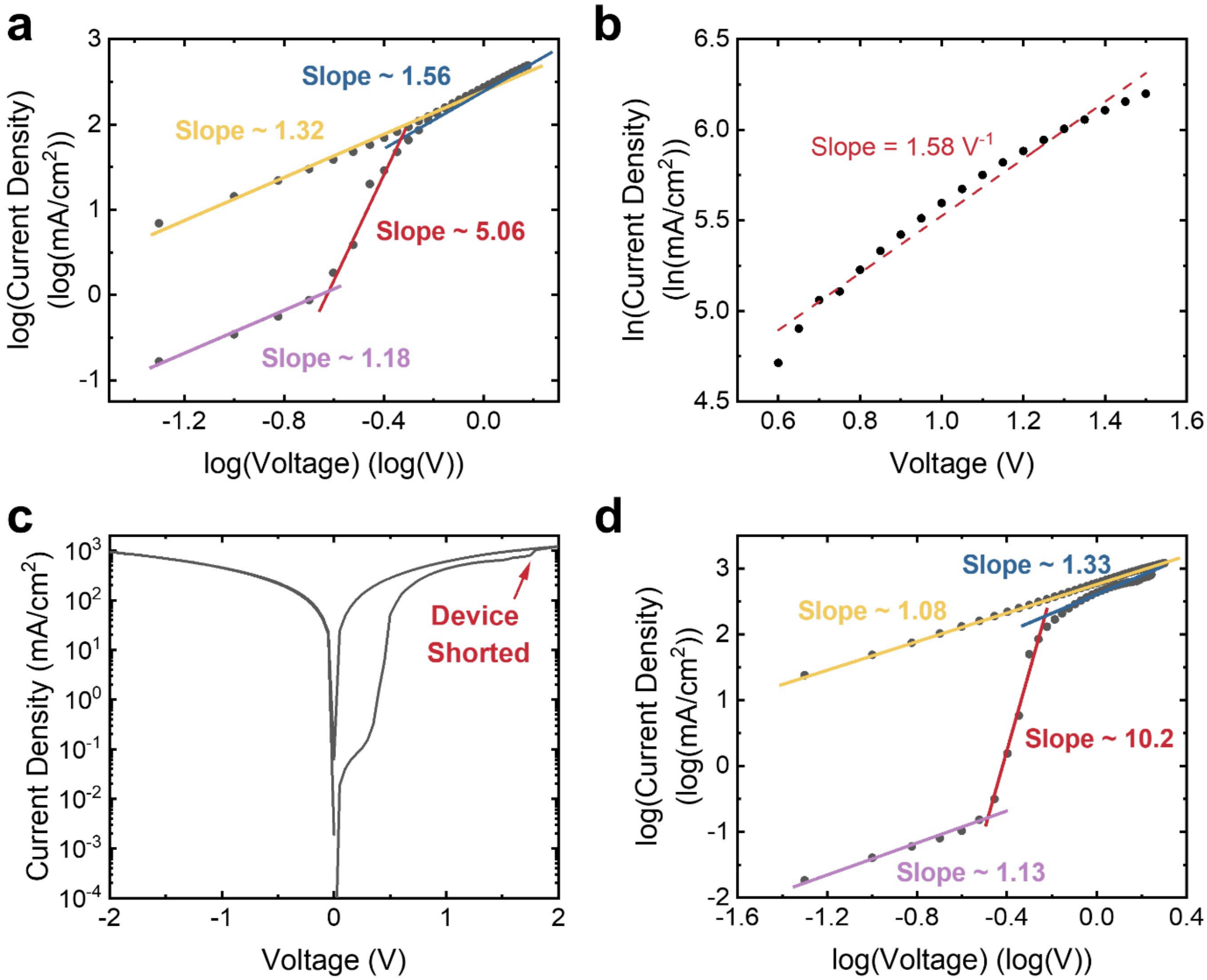

Figure S9. (a) Replotted *J-V* curve of the FTO/$MAPbI_3$/Ag memristor from Figure 2a on a double logarithmic scale. (b) Replotted high-voltage region of the *J-V* curve from Figure 2a, presented as the natural logarithm of the current density (*ln(J)*) as a function of the applied voltage (V_a). No strong linear dependence of *ln(J)* on V_a is observed. Even when the data are fitted based on the thermionic-emission model, the ideality factor is extracted to be 24.4, far exceeding the physically reasonable range for thermionic emission. Collectively, these observations indicate that thermionic emission is not the dominant charge transport mechanism in the high-voltage regime. (c) *J-V* characteristics of an FTO/$MAPbI_3$/Ag device that failed during a DC voltage sweep (0 → 2 → 0 → −2 → 0 V, 2250 mV/s). (d) Replotted *J-V* curve from (c) on a double-log scale.

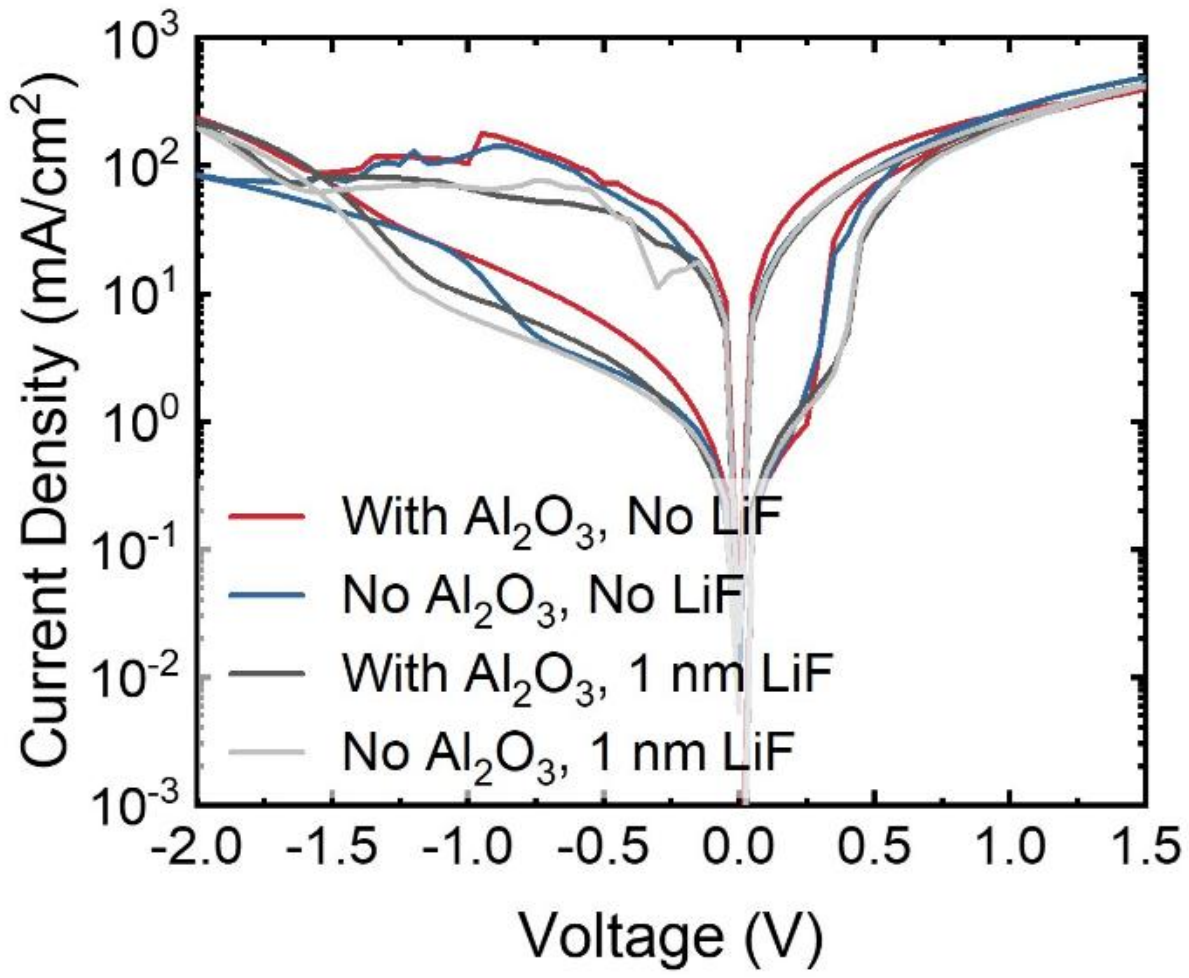

Figure S10. *J–V* characteristics of FTO/(10-ALD-cycle Al_2O_3)/$MAPbI_3$/(1 nm LiF)/Ag devices under a DC voltage sweep at a scan rate of 2250 mV/s. The introduction of the LiF interlayer results in a slightly higher turn-on voltage, which is likely attributed to the physical separation between the perovskite layer and the Ag electrode.

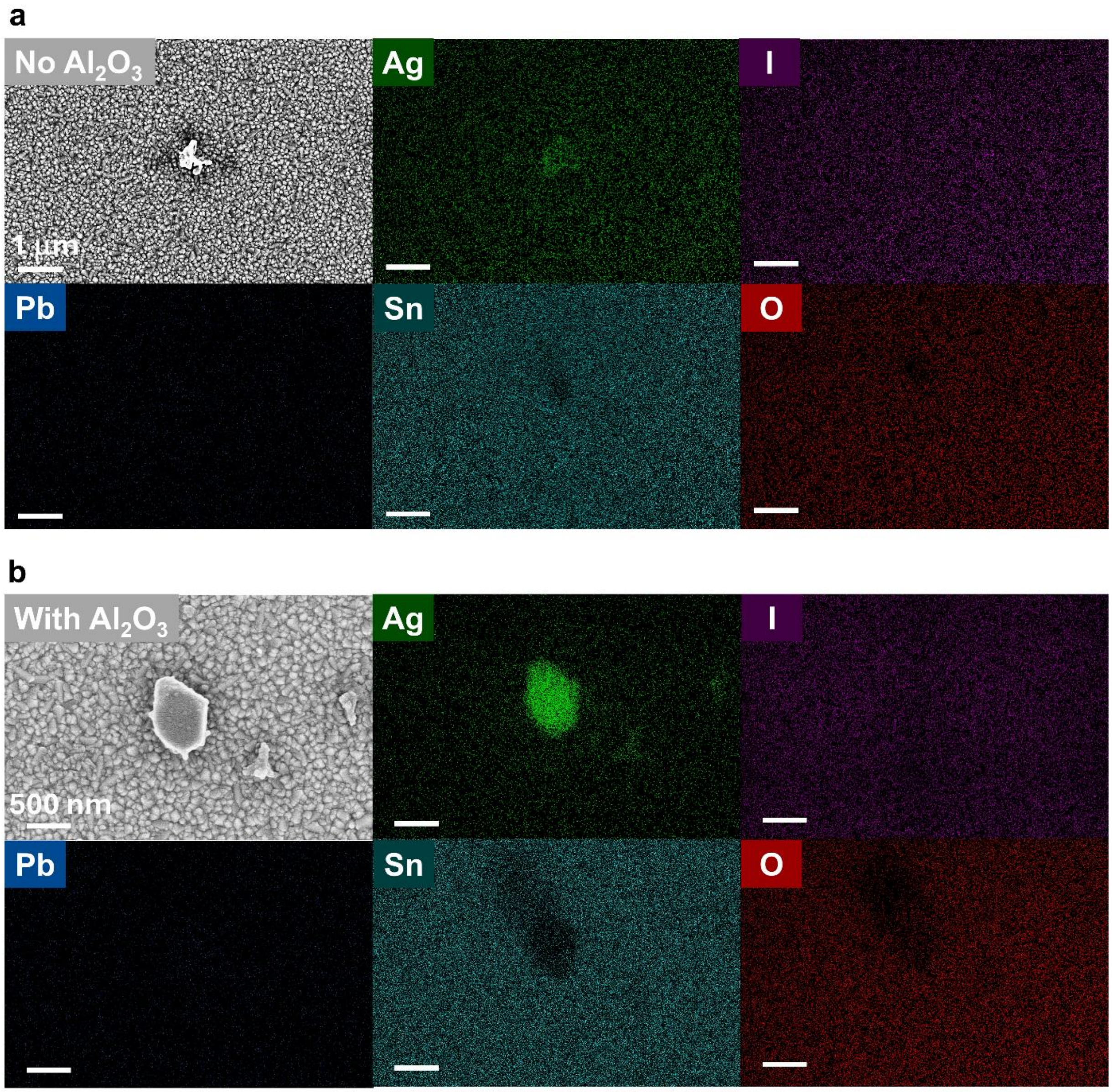

Figure S11. SEM images of Ag precipitates on (a) bare and (b) Al_2O_3-coated FTO substrate after cyclic switching, together with the corresponding EDX elemental mapping. Scale bars are 1 μm and 500 nm in (a) and (b) respectively. The absence of localized iodine signals within the regions of the precipitates indicates that the deposits are metallic Ag^0 rather than oxidized Ag species such as AgI.

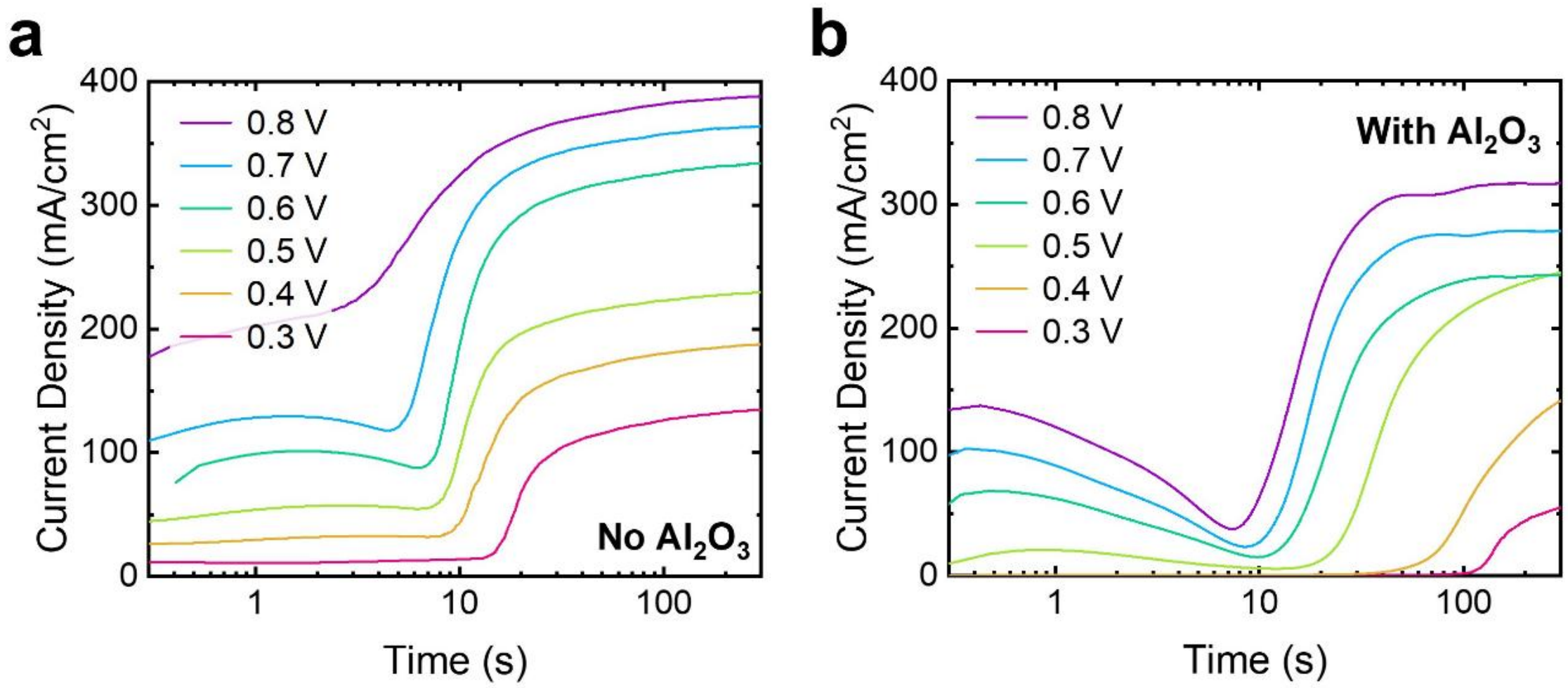

Figure S12. Current density transients during 5-min potentiostatic biasing of (a) FTO/$MAPbI_3$/LiF/Ag and (b) FTO/10-ALD-cyle Al_2O_3/$MAPbI_3$/LiF/Ag devices at indicated voltages.

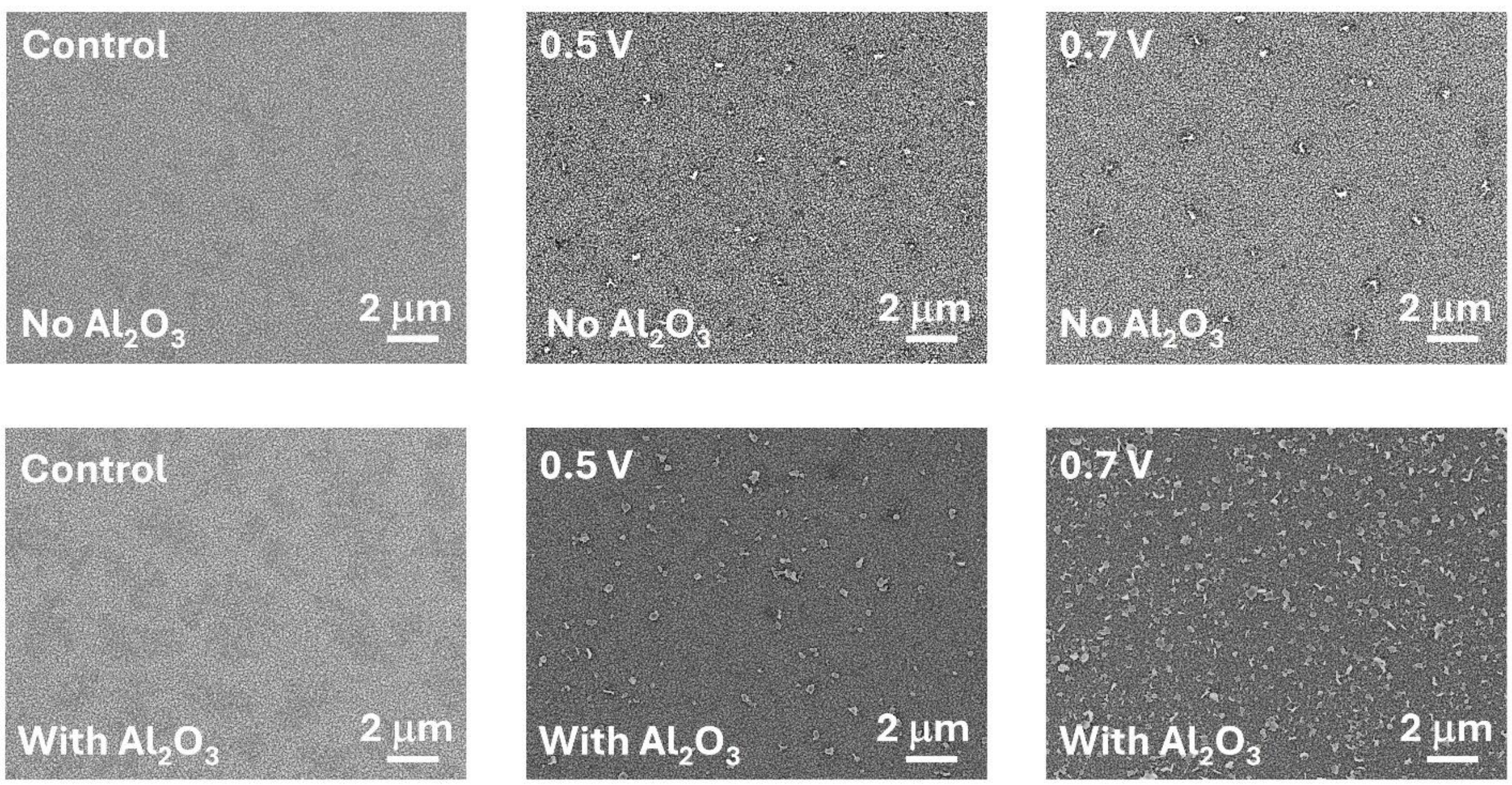

Figure S13. SEM images of bare and Al_2O_3-coated FTO substrates after perovskite removal, following potentiostatic biasing of FTO/(Al_2O_3)/$MAPbI_3$/LiF/Ag devices at different voltages. Images of bare and Al_2O_3-coated FTO surfaces from unbiased control devices are included for reference.

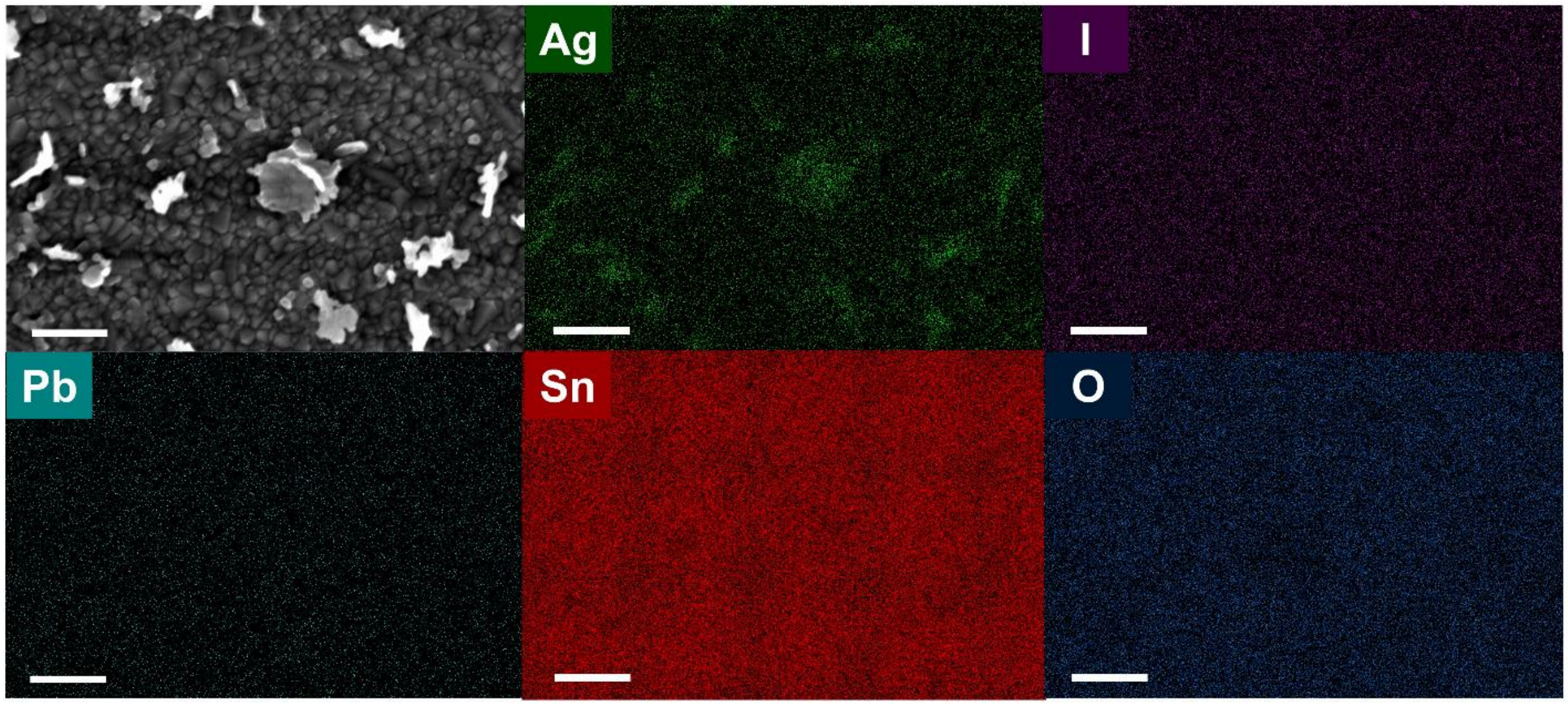

Figure S14. High-magnification SEM image of Ag precipitates on the Al_2O_3-coated FTO substrate featuring planar island growth, together with the corresponding EDX elemental mapping (scale bar: 500 nm). The absence of localized iodine signals within the regions of the precipitates indicates that the deposits are metallic Ag^0 rather than oxidized Ag species such as AgI.

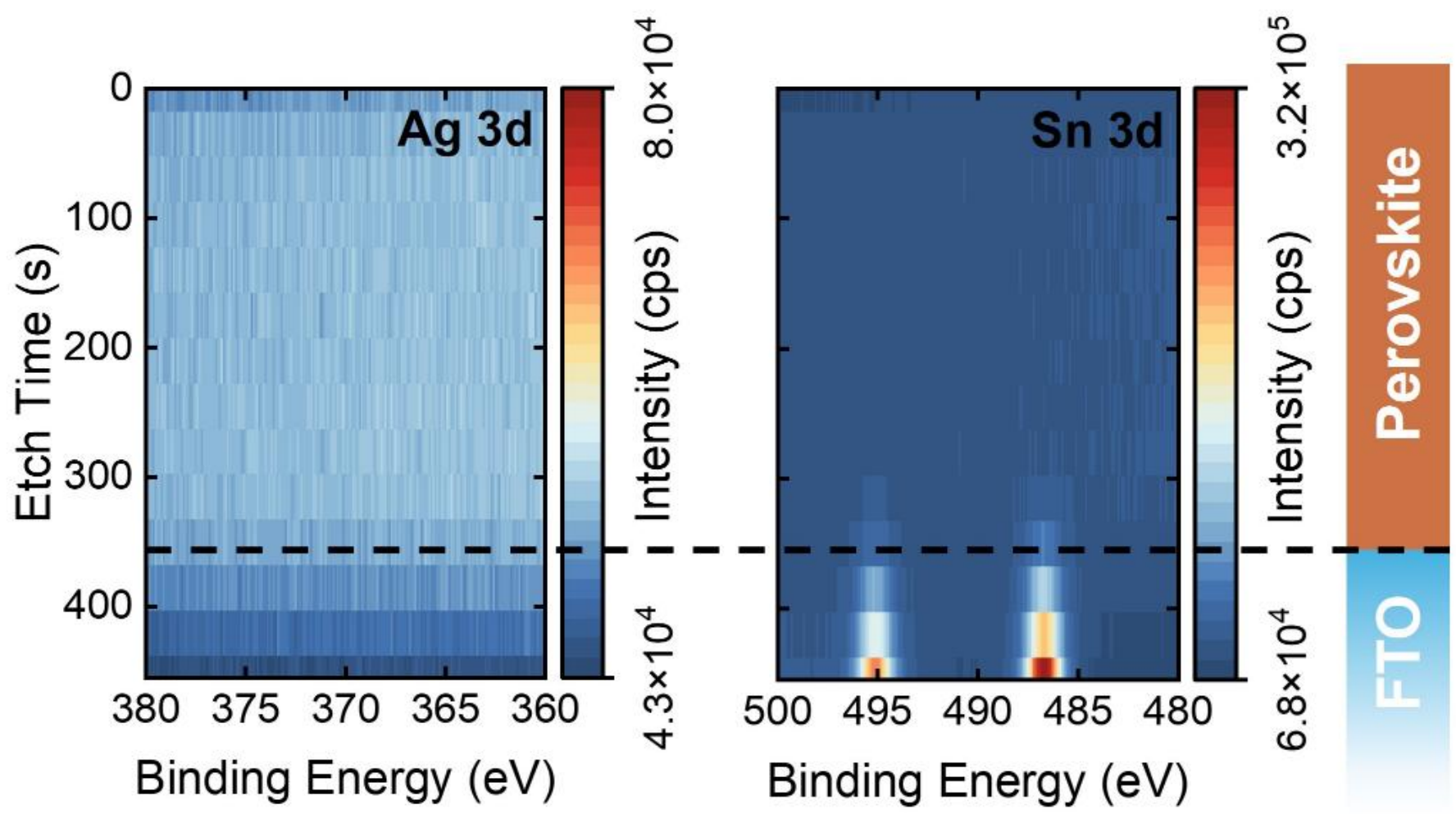

Figure S15. XPS depth profiles of Ag 3d and Sn 3d signals from an unbiased control device after Ag delamination.

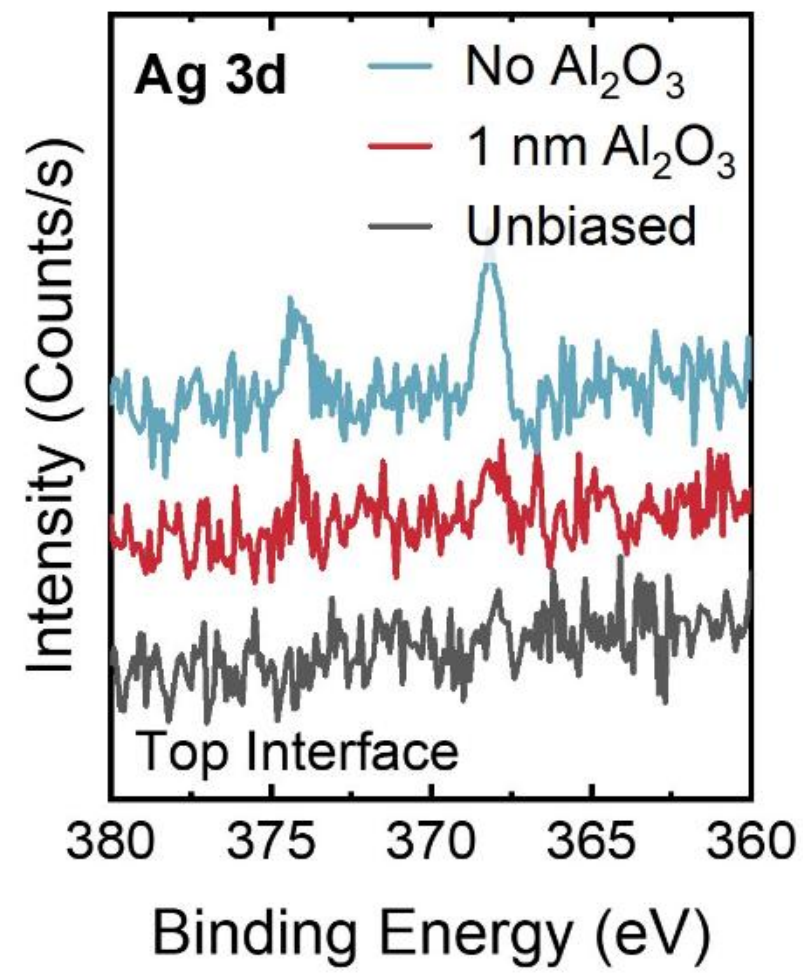

Figure S16. Ag 3d XPS spectra collected at the perovskite top interfaces of FTO/(Al_2O_3)/$MAPbI_3$/LiF/Ag devices after Ag delamination, following 5 min of biasing at 0.7 V, compared with an unbiased control device.

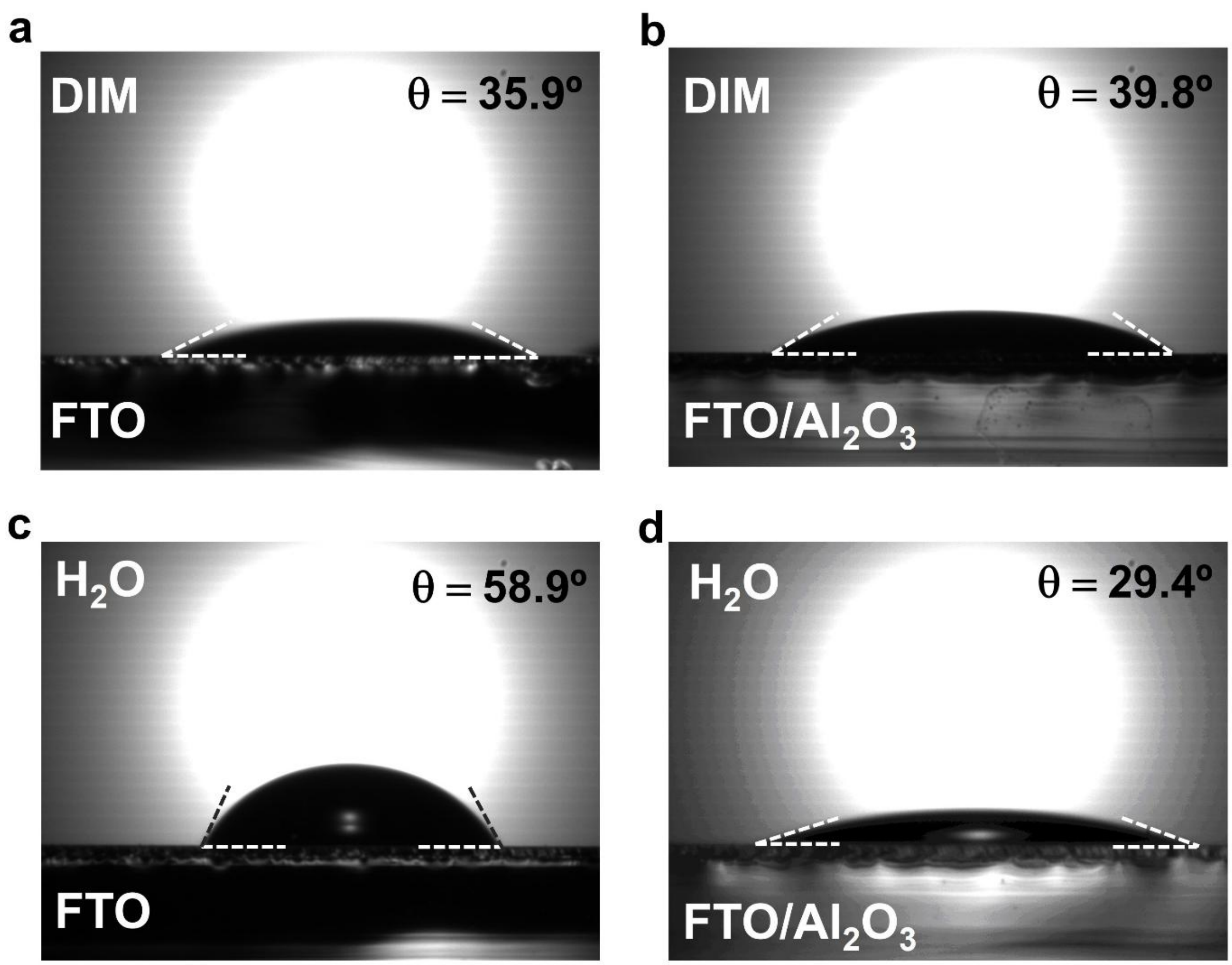

Figure S17. Contact angle measurements on (a, c) bare and (b, d) 10-ALD-cycle Al_2O_3-coated FTO substrates with (a, b) diiodomethane (DIM) and (b, d) water (H_2O) as the probe liquids.

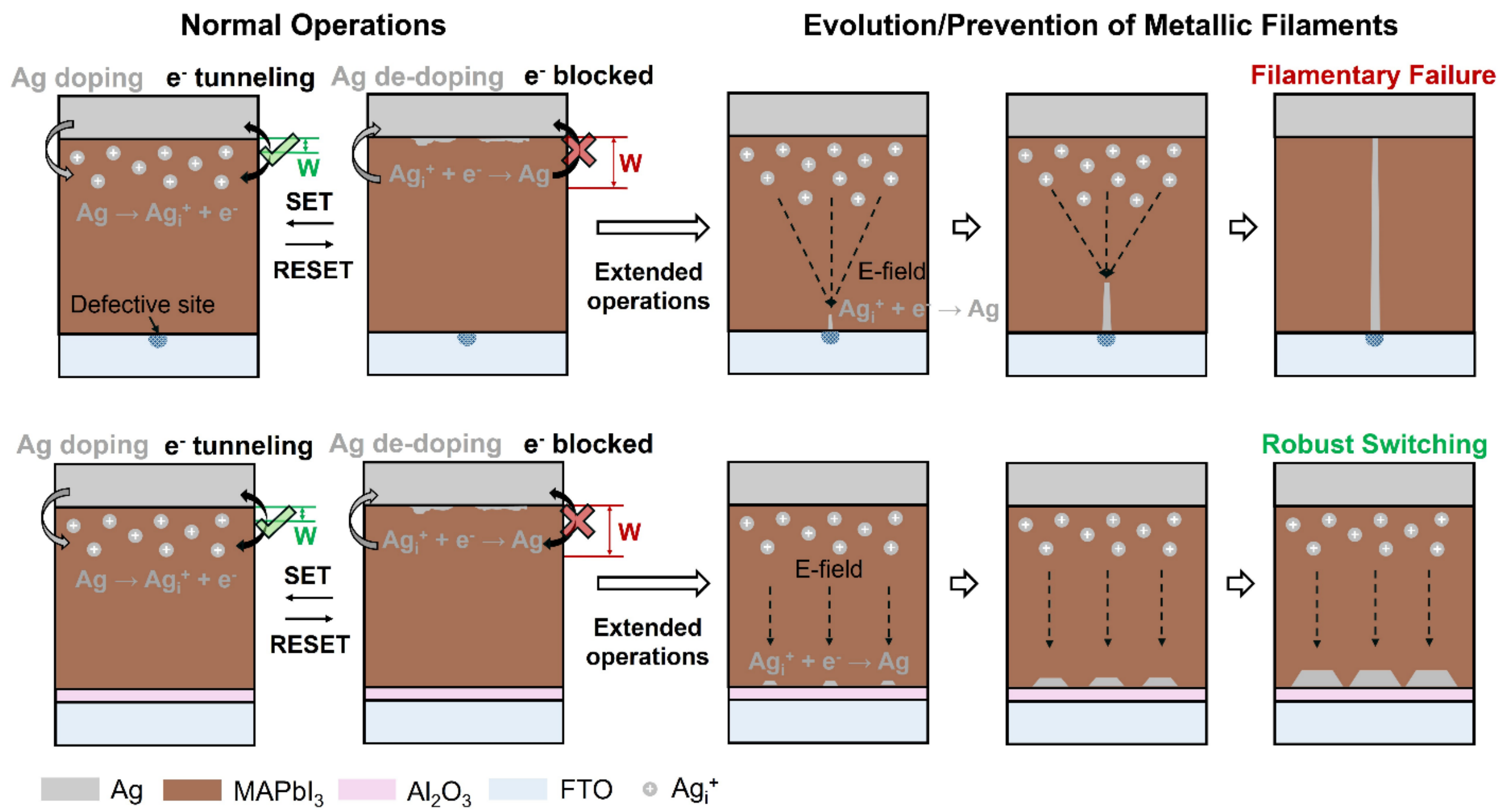

Figure S18. Full-device schematics illustrating the resistive switching and failure mechanisms in perovskite memristors with and without the Al_2O_3 interlayer. The schematics highlight the role of the Al_2O_3 interlayer in passivating the reactive defective sites on the FTO surface and maintaining a more uniform electric-field distribution across the $MAPbI_3$ layer. Note that the depletion width (W) at the $MAPbI_3$/Ag interface is intended only for qualitative comparison between the high- and low-resistance states and is not drawn to scale relative to the perovskite thickness.

Table S1. Summary of cyclic endurance and retention time of 3D perovskite-based memristors reported in recent years. Note that the operational V_{SET} and V_{RESET} represent the reported applied voltages during cyclic endurance tests.

Structure	Operational V_{SET}/ V_{RESET} (V)	Endurance (Cycles)	Retention (s)	Reference
ITO/PEDOT:PSS/$MAPbI_3$/Cu	+3/-3	3.0×10^3	3.0×10^4	[1]
Si/SiO_2/Ti/Pt/$MAPbI_3$/Ag	+0.15/-0.15	3.5×10^2	1.1×10^4	[2]
Si/SiO_2/Ti/Au/$MAPbI_3$/Ag	N/A	1.0×10^3	1.2×10^3	[3]
Si/SiO_2/Ti/Pt/$MAPbI_3$/Ag	+0.3/-0.7	1.3×10^3	3.0×10^4	[4]
ITO/$MAPbI_3$/Au	N/A	5.0×10^2	1.0×10^4	[5]
ITO/$MAPbBr_3$/Au	N/A	1.0×10^3	1.0×10^4	[6]
FTO/$MAPbI_{3-x}Cl_x$/Au	+0.13/-1.6	4.0×10^2	4.7×10^4	[7]
FTO/$MAPbI_{3-x}Cl_x$/Ag	+1.0/-2.5	1.0×10^4	2.0×10^3	[8]
Au/$MAPbI_3$/Au	N/A	1.0×10^3	1.0×10^4	[9]
ITO/$Cs_{0.06}FA_{0.78}MA_{0.16}Pb(I_{0.92}Br_{0.08})_3$/Au	+5/-5	1.0×10^3	1.0×10^5	[10]
ITO/Y:$MAPbI_3$/Al	N/A	3.0×10^3	1.0×10^4	[11]
ITO/$MAPbI_{3-x}Cl_x$/PEASCN/Al	N/A	1.0×10^3	1.0×10^4	[12]
Si/Pt/$FAPbI_3$/Ag	+0.25/-0.42	2.1×10^3	1.0×10^3	[13]
Pt/$MAPbBr_3$/TPBI/Ag	+0.8/-0.8	1.0×10^3	3.0×10^3	[14]
ITO/$MAPbI_3$/MoO_3/Ag	N/A	1.0×10^3	1.1×10^3	[15]
ITO/PTAA/$MAPbBr_3$/Graphite	+1.0/-2.5	1.0×10^3	1.0×10^4	[16]
ITO/$MAPbI_3$:Ag/Al	+1.7/-2.0	1.0×10^3	1.0×10^5	[17]
**FTO/Al_2O_3/$MAPbI_3$/Ag**	**+1.0/-2.0**	**1.5×10^4**	**1.0×10^5**	**This Work**

Table S2. Polar and dispersive surface energy components of probe liquids (γ_l^p and γ_l^d)[18] and the calculated polar and dispersive surface energy components of bare and Al_2O_3-coated FTO substrates (γ_s^p and γ_s^d). γ_l and γ_s stand for the total surface energies of the probe liquids and the substrates respectively.

	γ_l^p (mJ/m^2)	γ_l^d (mJ/m^2)	γ_l (mJ/m^2)
Water (H_2O)	51.0	21.8	72.8
Diiodomethane (DIM)	0.0	50.8	50.8
	γ_s^p (mJ/m^2)	γ_s^d (mJ/m^2)	γ_s (mJ/m^2)
FTO	12.3	41.6	53.9
FTO/Al_2O_3	29.4	39.7	69.1

Note S1. The Role of Iodide Species in the Resistive Switching Process.

Under the setting bias condition, iodide ions may accumulate near the anodically biased Ag contact, where they can potentially react with Ag to form AgI. Although the formation of AgI has been widely reported in perovskite-based memristors, the presence of AgI itself has been determined not to be responsible for resistive switching.[3, 19-21] Moreover, since AgI is known to be a solid-state Ag^+ conductor with a relatively small overpotential for the Ag^0/Ag^+ redox process, Ag^+ transport across the AgI interlayer is expected to remain both thermodynamically and kinetically favorable. Therefore, the presence of a AgI interlayer is unlikely to impede Ag^+ migration into the perovskite layer. In addition, considering the intrinsically thin nature of AgI and its similar electrical conductivity (≈10^{-7} S/cm) to that of $MAPbI_3$,[21] the presence of AgI is not expected to significantly influence charge-carrier transport across the $MAPbI_3$/Ag interface. Collectively, AgI is omitted from the switching-mechanism schematics (Figure 4) for clarity.

We also note that several previous studies on perovskite-based memristors proposed that AgI formation may increase the number of iodide vacancies (V_I^+) in the perovskite film, enabling accumulation of V_I^+ from the cathode to the anode until a through-film V_I^+ conductive filament is formed, thereby switching the device into its low resistance state (LRS).[3,19] However, in our study, experimental observations such as the gradual switching operation (Figure 2a), the area-dependent switching behavior (Figure 2c), and the quasi-ohmic conduction at the LRS (Figure S9a), strongly indicate an interface-type switching mechanism rather than a filamentary one. Accordingly, the formation of a conductive iodide-vacancy filament is unlikely in our devices and is therefore not considered as the switching mechanism. Together with the absence of non-volatile resistive switching in the Au-based devices (Figure S8), these results suggest that the redistribution of iodide species does not play a dominant role in the resistive switching behavior and is thus omitted from the mechanism schematics in Figure 4.

Note S2. Calculation of Substrate Surface Energies Based on Contact Angle Measurements.

Given the dispersive and polar components of the probe liquid surface energies (γ_l^d and γ_l^p, respectively) listed in Table S2, the dispersive and polar components of the substrate surface energy (γ_s^d and γ_s^p, respectively) can be calculated based on Fowkes surface energy theory:[22]

$$\gamma_l(1 + \cos\theta) = 2[(\gamma_l^d\gamma_s^d)^{1/2} + (\gamma_l^p\gamma_s^p)^{1/2}]$$

where γ_l is the sum of γ_l^d and γ_l^p and θ is the contact angle between the probe liquid and the substrate. According to the Fowkes theory, the total substrate surface energy, γ_s, is assumed to be the sum of the dispersive and polar components:

$$\gamma_s = \gamma_s^d + \gamma_s^p$$

Consequently, the γ_s^d, γ_s^p, and γ_s for both bare and Al_2O_3-coated FTO substrates are calculated and tabulated in Table S2.

References

1. Lin, G.; Lin, Y.; Cui, R.; Huang, H.; Guo, X.; Li, C.; Dong, J.; Guo, X.; Sun, B. An Organic–Inorganic Hybrid Perovskite Logic Gate for Better Computing. *J. Mater. Chem. C* **2015**, *3* (41), 10793–10798.
2. Choi, J.; Park, S.; Lee, J.; Hong, K.; Kim, D.-H.; Moon, C. W.; Park, G. D.; Suh, J.; Hwang, J.; Kim, S. Y.; Jung, H. S.; Park, N. G.; Han, S.; Nam, K. T.; Jang, H. W. Organolead Halide Perovskites for Low Operating Voltage Multilevel Resistive Switching. *Adv. Mater.* **2016**, *28* (31), 6562–6567.
3. Zhu, X.; Lee, J.; Lu, W. D. Iodine Vacancy Redistribution in Organic–Inorganic Halide Perovskite Films and Resistive Switching Effects. *Adv. Mater.* **2017**, *29* (29), 1700527.
4. Choi, J.; Le, Q. V.; Hong, K.; Moon, C. W.; Han, J. S.; Kwon, K. C.; Cha, P.-R.; Kwon, Y.; Kim, S. Y.; Jang, H. W. Enhanced Endurance Organolead Halide Perovskite Resistive Switching Memories Operable under an Extremely Low Bending Radius. *ACS Appl. Mater. Interfaces* **2017**, *9* (36), 30764–30771.
5. Heo, J. H.; Shin, D. H.; Moon, S. H.; Lee, M. H.; Kim, D. H.; Oh, S. H.; Jo, W.; Im, S. H. Memory Effect Behavior with Respect to the Crystal Grain Size in the Organic-Inorganic Hybrid Perovskite Nonvolatile Resistive Random Access Memory. *Sci. Rep.* **2017**, *7* (1), 16586.
6. Guan, X.; Hu, W.; Haque, M. A.; Wei, N.; Liu, Z.; Chen, A.; Wu, T. Light-Responsive Ion-Redistribution-Induced Resistive Switching in Hybrid Perovskite Schottky Junctions. *Adv. Funct. Mater*. **2018**, *28* (3), 1704665.
7. Zhou, F.; Liu, Y.; Shen, X.; Wang, M.; Yuan, F.; Chai, Y. Low-Voltage, Optoelectronic $CH_3NH_3PbI_{3-x}Cl_x$ Memory with Integrated Sensing and Logic Operations. *Adv. Funct. Mater*. **2018**, *28* (15), 1800080.
8. Shaban, A.; Joodaki, M.; Mehregan, S.; Rangelow, I. W. Probe-Induced Resistive Switching Memory Based on Organic-Inorganic Lead Halide Perovskite Materials. *Org. Electron.* **2019**, *69*, 106–113.
9. Kang, K.; Ahn, H.; Song, Y.; Lee, W.; Kim, J.; Kim, Y.; Yoo, D.; Lee, T. High-Performance Solution-Processed Organo-Metal Halide Perovskite Unipolar Resistive

Memory Devices in a Cross-Bar Array Structure. *Adv. Mater.* **2019**, *31* (21), 1804841.

10. Huang, Y.; Tang, L.; Wang, C.; Fan, H.; Zhao, Z.; Wu, H.; Xu, M.; Shen, R.; Yang, Y.; Bian, J. Triple-Cation Perovskite Resistive Switching Memory with Enhanced Endurance and Retention. *ACS Appl. Electron. Mater.* **2020**, *2* (11), 3695–3703.
11. Luo, F.; Ruan, L.; Tong, J.; Wu, Y.; Sun, C.; Qin, G.; Tian, F.; Zhang, X. Enhanced Resistive Switching Performance in Yttrium-Doped CH 3 NH 3 PbI 3 Perovskite Devices. *Phys. Chem. Chem. Phys.* **2021**, *23* (38), 21757–21768.
12. Pan, X.; Chen, X.; Duan, J.; Long, Y.; Wu, Y.; Tang, J.; Ma, G.; Zhang, J.; Wang, H. Three-Dimensional/Two-Dimensional Perovskite-Resistive Random-Access Memory with Low SET Voltage and High Stability. *ACS Appl. Electron. Mater.* **2023**, *5* (12), 6908–6917.
13. Lee, J.-H.; Yang, J.-M.; Kim, S.-Y.; Baek, S.; Lee, S.; Lee, S.-J.; Park, N.-G.; Lee, J.-W. Intact Metal/Metal Halide van Der Waals Junction Enables Reliable Memristive Switching with High Endurance. *Adv. Funct. Mater.* **2023**, *33* (14), 2214142.
14. Kim, H.; Kim, J. S.; Choi, J.; Kim, Y.-H.; Suh, J. M.; Choi, M.-J.; Shim, Y.-S.; Kim, S. Y.; Lee, T.-W.; Jang, H. W. $MAPbBr_3$ Halide Perovskite-Based Resistive Random-Access Memories Using Electron Transport Layers for Long Endurance Cycles and Retention Time. *ACS Appl. Mater. Interfaces.* **2024**, *16* (2), 2457–2466.
15. Luo, H.; Lu, L.; Zhang, J.; Yun, Y.; Jiang, S.; Tian, Y.; Guo, Z.; Zhao, S.; Wei, W.; Li, W.; Hu, B.; Wang, R.; Li, S.; Chen, M.; Li, C. In Situ Unveiling of the Resistive Switching Mechanism of Halide Perovskite-Based Memristors. *J. Phys. Chem. Lett.* **2024**, *15* (9), 2453–2461.
16. Fernandez-Guillen, I.; Aranda, C. A.; Betancur, P. F.; Vallés-Pelarda, M.; Momblona, C.; Ripolles, T. S.; Abargues, R.; Boix, P. P. Perovskite Thin Single Crystal for a High Performance and Long Endurance Memristor. *Adv. Electron. Mater.* **2024**, *10* (5), 2300475.
17. Yan, Y.; Ge, H.; Zhang, X.; Cui, C.; Cheng, J.; Li, X.; Liu, X.; Zhang, L.; Sun, Q. Nonlocalized Conductive Paths Construction and In-Depth Mechanism Analysis for the Robust Resistive Switching in Halide Perovskites. *Nano Lett.* **2025**, *25* (4), 1358–1366.

18. Kozbial, A.; Li, Z.; Conaway, C.; McGinley, R.; Dhingra, S.; Vahdat, V.; Zhou, F.; D'Urso, B.; Liu, H.; Li, L. Study on the Surface Energy of Graphene by Contact Angle Measurements. *Langmuir* **2014**, *30* (28), 8598–8606.
19. Sun, Y.; Tai, M.; Song, C.; Wang, Z.; Yin, J.; Li, F.; Wu, H.; Zeng, F.; Lin, H.; Pan, F. Competition between Metallic and Vacancy Defect Conductive Filaments in a $CH_3NH_3PbI_3$-based Memory Device. *J. Phys. Chem. C* **2018**, *122* (11), 6431-6436.
20. Gonzales, C.; Guerrero, A. Mechanistic and Kinetic Analysis of Perovskite Memristors with Buffer Layers: the Case of a Two-step Set Process. *J. Phys. Chem. Lett.* **2023**, *14* (6), 1395-1402.
21. Perez-Martinez, J. C.; Berruet, M.; Gonzales, C.; Salehpour, S.; Bahari, A.; Arredondo, B.; Guerrero, A. Role of Metal Contacts on Halide Perovskite Memristors. *Adv. Funct. Mater.* **2023**, *33* (47), 2305211.
22. Fowkes, F. M. Attractive Forces at Interfaces. *Ind. Eng. Chem.* **1964**, *56* (12), 40-52.
